# Combined Consideration of Tumor-Associated Immune Cell Density and Immune Checkpoint Expression in the Peritumoral Microenvironment for Prognostic Stratification of Non-Small-Cell Lung Cancer Patients

**DOI:** 10.3389/fimmu.2022.811007

**Published:** 2022-02-10

**Authors:** Yong Yang, Xiaobao Yang, Yichao Wang, Jingsong Xu, Hanyu Shen, Hongquan Gou, Xiong Qin, Gening Jiang

**Affiliations:** ^1^ Department of Thoracic Surgery, Shanghai Pulmonary Hospital, Tongji University School of Medicine, Shanghai, China; ^2^ Department of Laboratory Medicine, Ruijin Hospital, Shanghai Jiao Tong University School of Medicine, Shanghai, China; ^3^ Department of Oncology, Yueyang Hospital of Integrated Traditional Chinese and Western Medicine, Shanghai University of Traditional Chinese Medicine, Shanghai, China

**Keywords:** multiplex immunohistochemistry, immune checkpoint, tumor-associated immune cells, peritumoral and intratumoral microenvironment, non-small-cell lung cancer, clustering classification

## Abstract

Given the complexity and highly heterogeneous nature of the microenvironment and its effects on antitumor immunity and cancer immune evasion, the prognostic value of a single immune marker is limited. Here, we show how the integration of immune checkpoint molecule expression and tumor-associated immune cell distribution patterns can influence prognosis prediction in non-small-cell lung cancer (NSCLC) patients. We analyzed tissue microarray (TMA) data derived from multiplex immunohistochemistry results and measured the densities of tumor-infiltrating CD8+ and FOXP3+ immune cells and tumor cells (PanCK+), as well as the densities of programmed cell death 1 (PD-1)+ and programmed cell death ligand 1 (PD-L1)+ cells in the peritumor and intratumor subregions. We found a higher density of infiltrating CD8+ and FOXP3+ immune cells in the peritumoral compartment than in the intratumoral compartment. In addition, unsupervised hierarchical clustering analysis of these markers revealed that the combination of high CD8/FOXP3 expression, low PD-1 and PD-L1 immune checkpoint expression, and lack of epidermal growth factor receptor (EGFR) mutation could be a favorable predictive marker. On the other hand, based on the clustering analysis, low CD8/FOXP3 and immune checkpoint (PD-1 and PD-L1) expression might be a marker for patients who are likely to respond to strategies targeting regulatory T (Treg) cells. Furthermore, an immune risk score model was established based on multivariate Cox regression, and the risk score was determined to be an independent prognostic factor for NSCLC patients. These results indicate that the immune context is heterogeneous because of the complex interactions of different components and that using multiple factors in combination might be promising for predicting the prognosis of and stratifying NSCLC patients.

## Introduction

Lung cancer accounts for almost one-fifth of all cancer deaths worldwide (1). In the past decade, conventional treatments, such as resection, platinum-based chemotherapy, radiotherapy, and targeted therapy, have significantly improved the prognosis of non-small-cell lung cancer (NSCLC), which can have oncogenic driver mutations, such as epidermal growth factor receptor (EGFR) and anaplastic lymphoma kinase (ALK) mutations (2). However, only a portion of patients have these driver mutations, which are inevitably accompanied by acquired resistance. Immunotherapy based on immune checkpoints, such as programmed cell death 1 (PD-1) and programmed cell death ligand 1 (PD-L1), has recently offered a new approach to NSCLC treatment and has led to longer overall survival (OS) for some patients, especially those without sensitivity to therapies targeting mutations in oncogenic driver genes. Immune checkpoint inhibitors (ICIs), including pembrolizumab, nivolumab, atezolizumab, and durvalumab, have been approved by the United States FDA to treat lung cancer (3, 4). However, only approximately 20% of NSCLC patients benefit from ICIs.

The basic principle of immunotherapy is to regulate tumor-immune interactions. Many reports have shown the genetic, epigenetic, and transcriptomic characteristics of NSCLC patients (5–7), but the understanding of the immune microenvironment in NSCLC is still incomplete. According to the density and distribution of CD8+ T cells and FOXP3+ regulator T (Treg) cells etc, which determine the classification of “hot” versus “cold” tumors, the clinical outcome of various cancer patients can be predicted: “hot” tumors have potential sensitivity to immunotherapy (8). The utility of CD8+ tumor-infiltrating lymphocytes (TILs) or FOXP3+ TILs as independent prognostic factors in NSCLC patients is controversial (9). Therefore, it is important to investigate the balance between CD8+ TILs and FOXP3+ TILs in the tumor microenvironment. The prognostic utility of the CD8/FOXP3 ratio in the tumor microenvironment has been reported in various cancer types, including NSCLC (10–12). However, the association between the preexisting CD8/FOXP3 ratio in the tumor microenvironment and the outcome of immunotherapy is still not clear. Given that PD-1 and PD-L1 have served as therapeutic targets in clinical practice, combining these two markers with CD8/FOXP3 could guide the classification and immunotherapy of patients in a superior manner.

According to the PD-L1 status and the number of TILs, tumors can be divided into four categories (13): type I adaptive immune resistant (PD-L1 positivity with a high number of TILs), type II immune ignorant (PD-L1 negativity with a low number of TILs), type III intrinsic induction (PD-L1 positivity with a low number of TILs), and type IV immune tolerant (PD-L1 negativity with a high number of TILs). However, a detailed tissue characterization that combined PD-1, PD-L1, CD8 and FOXP3 expression levels indicated the immune-mediated tumor growth and regression were only partially investigated in NSCLC (14).

In recent years, characterization of the TIL type, density, and spatial distribution in the local tumor microenvironment has provided a foundation for the development of immunotherapy for NSCLC patients, and several new technologies that preserve the spatial organization have been developed to help evaluate multiple markers (15). These methods can better identify immune biomarkers in the tumor immune microenvironment for prognostic stratification of NSCLC patients. In fact, the accuracy of multiplex immunohistochemistry IHC (mIHC)/immunofluorescence (IF) testing has been shown to be better than that of PD-L1 expression and gene expression characteristics in predicting the response of multiple tumor types to PD-1 checkpoint blockade (16).

In the present study, the expression of four key immune markers, the checkpoint molecules PD-1 and PD-L1, the antitumor T cell marker CD8 and the immunosuppressive Treg marker FOXP3 and their potential prognostic value in NSCLC were investigated. More specifically, formalin-fixed paraffin-embedded (FFPE) tumor tissue microarrays (TMAs) representing 98 NSCLC patients were assessed using mIHC techniques to explore the role of these markers in patients. The data were used to assess disease prognosis and patient survival and stratification. We found a high density of infiltrated CD8+ and FOXP3+ immune cells in the peritumoral component compared to the intratumoral component, and the combination of subregion-specific infiltration patterns of immune cells with PD-1 and PD-L1 expression could be used to predict patient prognosis. Unsupervised hierarchical clustering analysis of the expression of CD8/FOXP3 and the PD-1 and PD-L1 immune checkpoints was performed, and the results revealed that stratification of patients based on the expression of these markers might guide anti-PD-1/PD-L1 therapy or Treg cell-targeting strategies.

## Materials and Methods

### Patient Cohort and Tissue Microarray Preparation

NSCLC tissues were obtained from Shanghai Jiao Tong University, Ruijin Hospital and Tongji University, Shanghai Pulmonary Hospital. The use of human specimens was approved by the Shanghai Jiao Tong University Human Ethics Committees, and informed consent was obtained from the patients involved in this study. The tissues were formalin-fixed and paraffin-embedded. H&E staining was performed on these sections, and the results for each section were reviewed by an independent surgical pathologist. A TMA of 180 tissues was constructed based on the H&E staining results, including 82 paired tumor and adjacent normal tissue samples (164 tissue samples), as well as another 16 NSCLC tumor tissue samples. However, some of the tumor tissue and adjacent normal tissue samples were excluded due to incompleteness. In total, 97tumor and 81 adjacent normal tissue samples were used in this study. The detailed clinical information of the patients is shown in **Supplementary Table S1** and the basic characteristics of the patients is shown in **Supplementary Table S2**. H&E staining of the TMA was performed to further validate the pathological diagnosis of each sample (**Supplementary Figure S1**). The core diameter of each sample in the TMA was 1.5 mm.

### mIHC

Multiplexed immunohistochemical staining was based on the fluorescent tyramine signal amplification (TSA) reagents, which allow antigen retrieval to remove primary and secondary antibodies, while the fluorescence signal can be retained until all antigens acquire their respective fluorophores (17). Paraffin-embedded sections were deparaffinized with xylene and gradient ethanol solutions, and antigen retrieval was conducted *via* microwave treatment. Endogenous peroxidase was neutralized with endogenous peroxidase blocking solution (Beyotime, Shanghai, China), and Opal antibody diluent/block (Akoya Biosciences, MA, United States) was used to block the binding of irrelevant antibodies. The primary antibodies included anti-CD8α (clone D8A8Y, dilution 1:500, Cell Signaling, MA, United States), anti-FOXP3 (clone D2W8E^™^, dilution 1:100, Cell Signaling), anti-PD-1 (clone D4W2J, dilution 1:250, Cell Signaling), anti-PD-L1 (clone E1L3N^®^, dilution 1:350, Cell Signaling) and anti-pan-keratin (clone C11, dilution 1:50, Cell Signaling) antibodies. Following application of the Opal polymer HRP anti-mouse/rabbit secondary antibodies (Akoya Biosciences), to detect the antibody staining, the tissues were incubated with one of the following fluorophores according to the manufacturer’s instructions: Opal Polaris 520, Opal Polaris 570, Opal Polaris 620, Opal Polaris 690 or Opal Polaris 780 (dilution 1:100). The tissue chip was then mounted in ProLong Gold Antifade Reagent with DAPI (Cell Signaling). Whole slide tissue scanning was performed at 20× magnification using the Vectra Polaris System (Akoya Biosciences) to capture the staining image. To further analyze the spatial distribution of CD8+ and FOXP3+ cells in the area surrounding the tumor, an algorithm was designed to create a 100-μm-thick band outside the tumor margin. The peritumoral compartment was defined as the region outside the tumor margin within 100 μm.

### Data Analysis

Spectral libraries were generated using single-stain scans, and according to the manufacturer’s instructions, image deconvolution was performed with inForm software (v2.4.8; Akoya Biosciences). The settings of the training images were applied to batch analysis for all scanned images. Data tables exported from inForm 2.4.8. were employed using Rstudio (Rstudio v3.6.1) for further analyses. The R packages “Phenoptr” and “PhenoptrReports” were used according to the manufacturer’s instructions (Phenoptics™, Akoya), and combined results for cell counts, cell percentages and cell densities were exported for further graphic generation.

### RNA Sequencing and Clinical Information From TCGA

In this study, mRNA expression data and clinical information were downloaded from the NCI Genomic Data Commons (GDC) portal (https://portal.gdc.cancer.gov). The raw RNA-seq count matrix and clinical information were obtained using the TCGAbiolinks package in R (v3.6.1). The count matrix was transformed into reads per kilobase million (FPKM). FPKM values for CD8A (CD8), FOXP3, PDCD1 (PD-1) and CD274 (PD-L1) RNA were extracted to verify the risk score model constructed from the TMA results.

### Hierarchical Clustering Analysis

Hierarchical clustering was used to cluster NSCLC patients according to the TMA results. The expression and ratios were log2 transformed, and hierarchical clustering was performed in R with Euclidean distances using the ward.D2 method.

### Statistical Analysis

Statistical analysis was performed using GraphPad Prism 8.0.2 software (GraphPad, Inc., San Diego, CA, USA). Two-tailed t tests were used to determine statistically significant differences in unpaired data. Paired t tests were used to assess differences in paired data. The Spearman correlation coefficient was used to assess correlations among different marker expression levels. Univariate and multivariate Cox regression models were used to analyze the independent prognostic factors and immune markers. Overall survival among subgroups was estimated using the Kaplan–Meier method, and significant differences were assessed using log-rank tests. P < 0.05 was defined as statistically significant.

## Results

### Exploration of NSCLC Phenotypes Using mIHC

**Figure 1:**
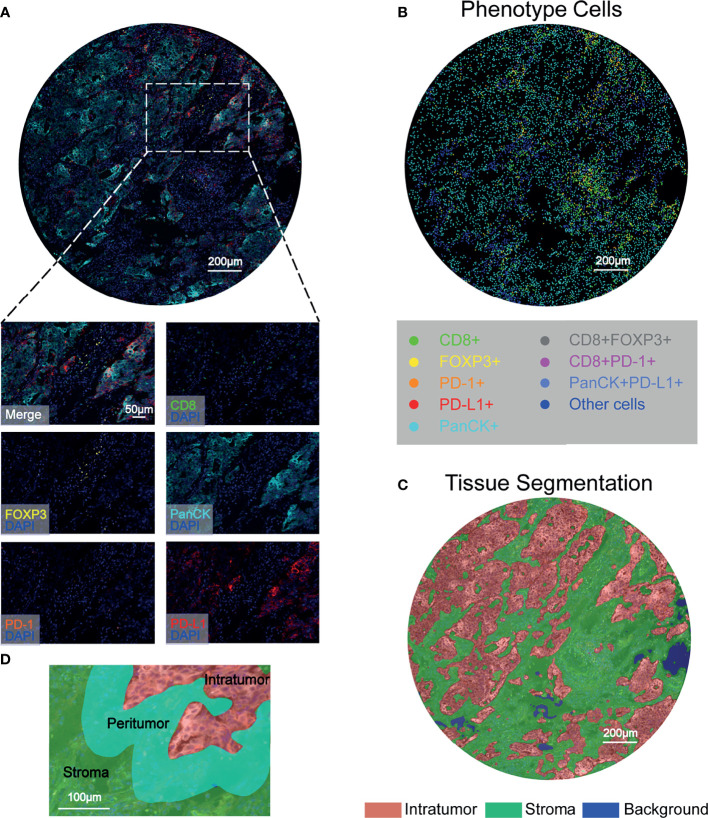
Characterization of tumor-associated immune cells and immune checkpoints in NSCLC tissues using six-color mIHC. **(A)** Digital scanning produced a multispectral image (MSI) of one TMA core from NSCLC patient tissues. **(B)** Corresponding identification markers employed in this study. Representative images were acquired with a Vectra Polaris microscope and show multiple staining patterns in NSCLC tissues. PanCK, as a tumor cell marker, is shown in cyan; FOXP3+ T cells are shown in yellow; and PD-1+ cells are shown in orange. Red indicates PD-L1+ cells, and green indicates CD8+ cells. The multiplexed images show colocalization of different markers, such as CD8 and PD-1. Scale bar: 200 μm. **(C)** Segmentation of the tumor compartment into intratumor (red) and stromal (green) tissues, which were distinguished by PanCK+ cells. Scale bar: 200 μm. **(D)** Representative image of the peritumoral regions, which were selected for CD8+, FOXP3+, PD-1+ and PD-L1+ cell quantification.

To characterize the resident and infiltrating immune cell landscape in NSCLC in situ, a workflow was established and optimized for mIHC to assess five different markers to simultaneously depict CD8+ T cells, FOXP3+ Treg cells, PD-1+ cells, and PD-L1+ cells. PanCK positivity was used to define the tumor component. After spectral separation using inForm software, the image was separated into its inherent fluorophores, and the corresponding image was visualized (**Figure 1A**). We relied on 6-color mIHC staining of TMA samples to evaluate the densities of CD8+ T cells, FOXP3+ Treg cells, and PD-1+ and PD-L1+ cells in tumor tissues and adjacent normal tissues (**Figure 1B**). Then, inForm was used to assess phenotype in both the tumor tissue compartment and stromal tissue compartment, where the tumor was defined by a machine learning algorithm that identifies PanCK-positive regions as the tumor tissue compartment (**Figure 1C**). An algorithm was designed based on pattern recognition that quantified immune cells within two tumor compartments: the peritumoral region related to the invasive tumor margin (stromal-tumor edge) and the intratumoral region was the area within the tumor. The infiltrating immune cells in the peritumoral and intratumoral regions were investigated in patients with NSCLC (**Figure 1D**). Therefore, the levels of immune cells and checkpoints were evaluated in different microdissection subregions.

### Correlation of a High Density of Tumor-Associated Immune Cells With an Immunosuppressive Phenotype in NSCLC

**Figure 2:**
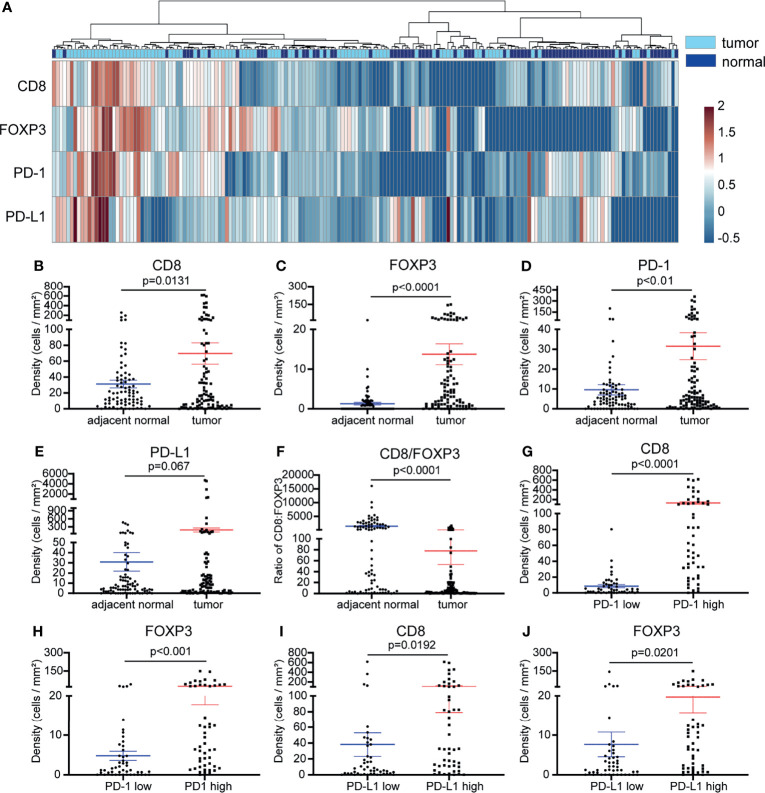
Comparison of the cell densities of CD8+ T cells, FOXP3+ cells, PD-1+ cells and PD-L1+ cells in tumor and adjacent normal tissues and the relationships between these markers. **(A)** Heatmap representation of the hierarchical clustering showing the density levels of CD8+, FOXP3+, PD-1+ and PD-L1+ cells and a dendrogram of the results of the unsupervised hierarchical clustering of tumors and adjacent normal samples. **(B–D)** Compared with those in adjacent normal samples, the densities of CD8+ cells, FOXP3+ Treg cells, and PD-1+ cells in tumor samples were significantly increased (p <0.01-0.0001). **(E)** Compared with that in adjacent normal tissues, the expression of PD-L1 was inclined to increase in tumor tissues (p =0.067). **(F)** Compared with that in adjacent normal tissues, the ratio of CD8/FOXP3 in tumor tissues was significantly reduced (p <0.0001). **(G–J)** The CD8+ T cell and FOXP3+ Treg cell densities were significantly higher in NSCLC tissues with high PD-1+ cell density **(G, H)** and in the PD-L1 high expression group **(I, J)** (p < 0.05-0.0001). Error bars represent the SEM.

We initially compared the densities of CD8+ T cells, FOXP3+ Treg cells, PD-1+ and PD-L1+ cells between tumor tissue and paired adjacent normal tissues. We were able to divide the samples into two groups based on their origin (tumor or adjacent normal tissue) (**Figure 2A**). We found that the densities of CD8+, FOXP3+ and PD-1+ cells were increased in the tumor tissue (p <0.01) (**Figures 2B**–**D**), and there was no significant difference in the levels of PD-L1+ cells between the tumor and adjacent normal tissues. (**Figure 2E**). Next, we evaluated the CD8+ to FOXP3+ cell ratio. Compared with tumor tissue, adjacent normal tissue showed a significantly higher CD8/FOXP3 ratio (**Figure 2F**). We further analyzed the relationships between CD8+ T cells, FOXP3+ Treg cells, PD-1+ cells and PD-L1+ cells in NSCLC. PD-1+ or PD-L1+ cells inhibit T cell function and lead to suppression of local antitumor immunity. Their high levels are often closely related to a poorer prognosis and poorer patient survival in many cancer types, including NSCLC (18). Therefore, we set out to determine whether there was any relationship between the levels (density of positive cells per square millimeter or expression intensity per square millimeter) of cells expressing PD-L1, PD-1, CD8, and FOXP3. We first divided the NSCLC samples into two groups based on the median PD-1+ cell density. We next compared the density of CD8+ and FOXP3+ cells between the two groups. The density of CD8+ T cells and FOXP3+ Treg cells was significantly higher in the high PD-1 expression group (**Figures 2G, H**). As shown in **Supplementary Figure S2A**, the densities of CD8+ cells and FOXP3+ Treg cells in NSCLC tissue were significantly positively correlated (r = 0.3691, p <0.0001), and the densities of CD8+ T cells and FOXP3+ Treg cells were positively correlated with PD-1 expression (r = 0.7234, p <0.0001 and r = 0.4684, p <0.0001, respectively) (**Supplementary Figures S2B, C**). In addition, when we divided the NSCLC samples into two groups based on the PD-L1 expression level, we observed significantly higher CD8+ T cell and FOXP3+ Treg levels in the high PD-L1 expression group (**Figures 2I, J**). Together, these results showed that there was a correlation between high PD-1/PD-L1 expression and high CD8+ and FOXP3+ cell infiltration. This indicates that in the case of an immune-resistant subtype we are likely to find high CD8+ T cell infiltration, leading to increased Treg infiltration and to higher PD-1/PD-L1 expression induced in an adaptative manner by the cancer.

### NSCLC Samples From Peritumor and Intratumor Subregions Show Distinct Immune Subsets and Checkpoint Signatures

**Figure 3:**
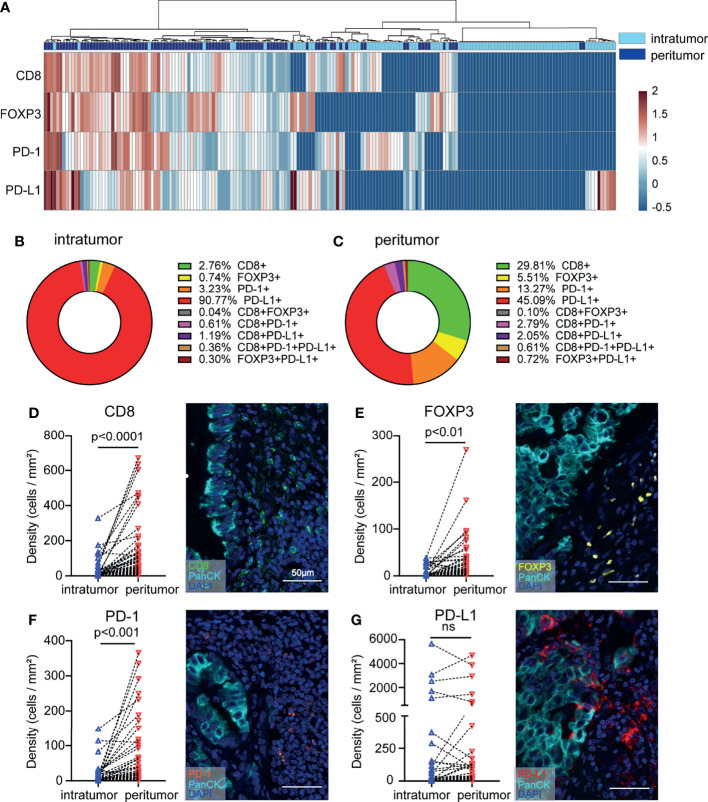
NSCLC tissue samples display increased infiltration of heterogeneous T cell subpopulations in the peritumoral subregion. **(A)** Heatmap representation of the results of the hierarchical clustering showing the densities of CD8+, FOXP3+, PD-1+, and PD-L1+ cells and a dendrogram of the results of the unsupervised hierarchical clustering of peritumoral and intratumoral tissues. **(B, C)** The distribution of each immune cell subpopulation in peritumoral and intratumoral tissues. **(D–G)** Differences in immune cell density according to the sampling strategy. The y-axis represents each immune cell density. The x-axis of each point is labeled with the peritumoral and intratumoral sampling strategy. Lines connect the two points of the densities of each marker relating to the same tumor. Representative images are shown on the right. Paired t tests were used to assess differences, and p values are reported.

Due to tumor distribution heterogeneity, the prognostic value of immune variables must be analyzed separately in different subregions. We investigated whether the density levels of CD8+ T cells, FOXP3+ Tregs, PD-1+ cells, and PD-L1+ cells could refine NSCLC patient subgroups. Heatmap and cluster analyses were performed to assess the potential correlation between different NSCLC samples from the surrounding and intratumor subregions. We were able to distinguish the unique immune subregions between peritumoral and intratumoral tissues according to the levels of CD8+, FOXP3+, PD-1+ and PD-L1+ cells (**Figure 3A**). Direct comparisons between the peritumor and intratumor subregions in patient samples are presented, and the proportions of T cell subpopulations were analyzed (**Figures 3B, C**). By comparing the immune subsets and checkpoints between peritumoral and intratumoral tissues from the same patient, a significant increase in the proportions of CD8+, FOXP3+, and PD-1+ T cell populations was identified in the peritumoral region (P < 0.01-0.0001) (**Figures 3D**–**F**). In contrast, the proportion of PD-L1+ cells was not significantly different between the peritumoral and intratumoral subregions (**Figure 3G**). The higher number of cytotoxic T cells in the peritumor tissue might represent a reaction at the edge of the tumor, which led us to further investigate peritumoral subregions.

### Clustering Analysis Confirms the Favorable Prognostic Value of the Combined Consideration of Immune Subsets and Checkpoints in NSCLC

**Figure 4:**
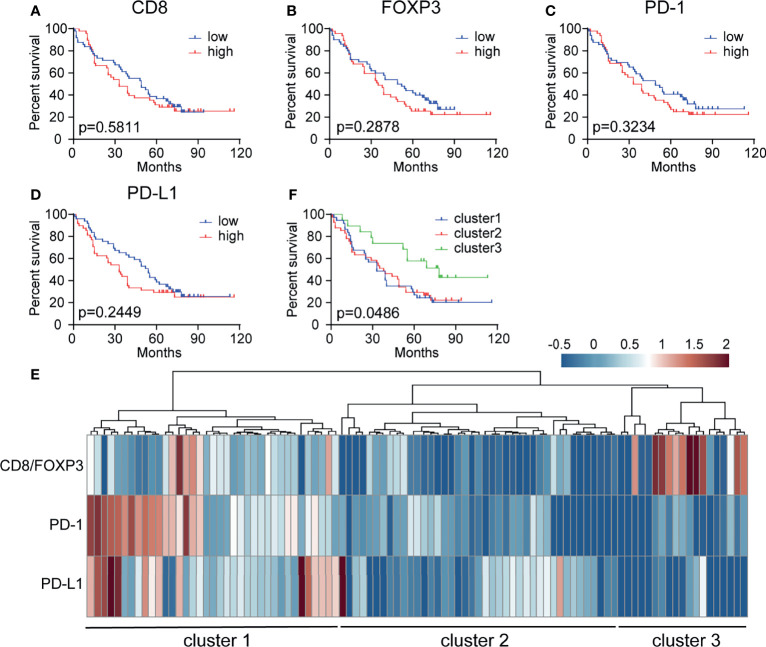
Patient stratification based on CD8+, FOXP3+, PD-1+ and PD-L1+ cell density has prognostic value. **(A–D)** Kaplan–Meier curve showing the prognostic effect of CD8, FOXP3, PD-1, and PD-L1 expression levels on overall survival (OS) in 97 NSCLC patient samples. The median cutoff value was used to distinguish between the high and low groups. A log-rank test was used to determine significance. **(E)** Patient stratification based on hierarchical clustering of the CD8/FOXP3 ratio, PD-1+ cells, and PD-L1+ cells in the peritumoral subregion. **(F)** Kaplan–Meier curve illustrating the prognostic effect on OS in three groups of patients stratified based on the CD8/FOXP3 ratio and checkpoint marker expression [based on data from panel **(E)**].

We next evaluated the different prognostic implications of the densities of CD8+, FOXP3+, PD-1+ and PD-L1+ cells in the peritumor subregions. Kaplan–Meier analysis of the entire patient cohort showed that the individual densities of CD8+ T cells, FOXP3+ Treg cells, PD-1+ cells, and PD-L1+ cells had no significant prognostic value for patient survival (**Figures 4A**–**D**). Various studies have proposed that tumors can be divided into different subtypes according to the density and spatial distribution of immune subpopulations in peritumor or intratumor subregions (19). Here, unsupervised clustering based on immune cell density identified three clusters with significantly different survival rates, among which the OS of cluster 3 was significantly better than that of clusters 1 and 2 (P = 0.0486) (**Figures 4E, F**). Cluster 3 may have a high CD8+ cell proportion and low PD-1/PD-L1+ cell proportions, which are associated with a better prognosis. In addition, we speculate that cluster 2 has a poor prognosis, but this cluster may improve CD8+ cell function through Treg cell targeting. Cluster 1 may require anti-PD-1/PD-L1 treatment.

### High CD8/FOXP3 and High PD-L1 Expression Are Correlated With Better Survival Outcomes in Subgroup Patients

**Figure 5:**
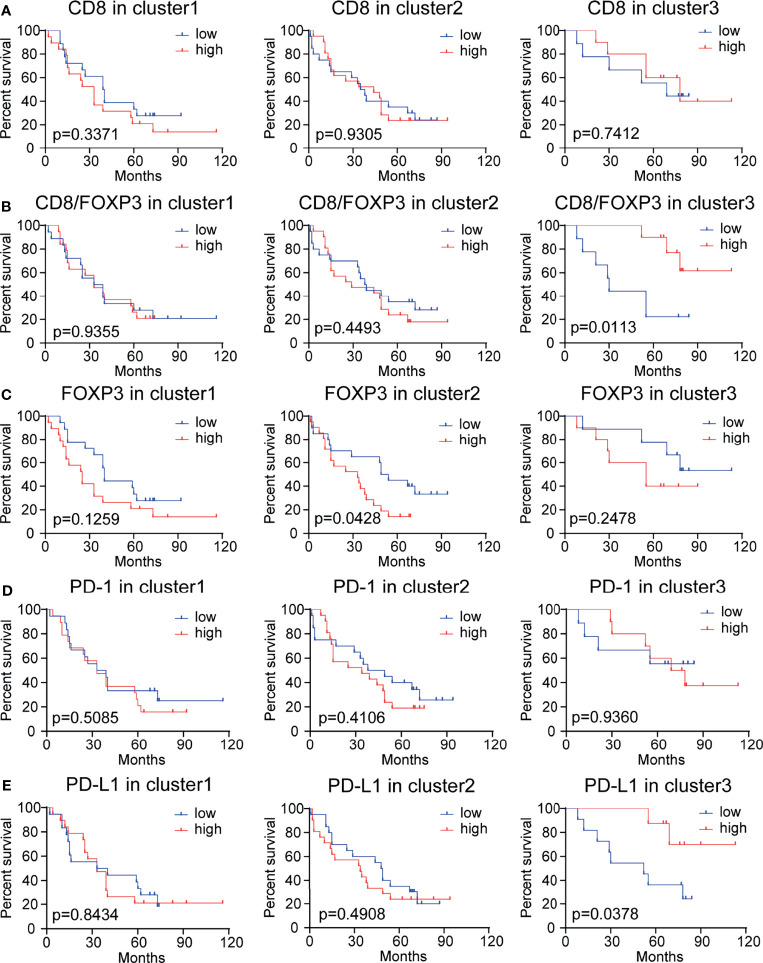
Kaplan–Meier analysis of the overall survival rate (OS) in subgroups of patients stratified based on CD8, CD8/FOXP3, FOXP3, PD-1, and PD-L1 expression levels. **(A)** Survival results for 97 NSCLC patients with different CD8+ cell densities. These patients were sorted into 3 clusters generated based on the CD8/FOXP3 ratio and PD-1 and PD-L1 expression levels. Patients with high or low CD8 expression had no significant difference in OS (p >0.05). **(B)** Survival results for 97 NSCLC patients with different CD8/FOXP3 ratios. The survival rate of patients with higher CD8/FOXP3 expression in cluster 3 was significantly increased (p <0.05). **(C)** Survival results for 97 NSCLC patients with different FOXP3 expression levels. Patients with higher FOXP3 expression in cluster 3 had poor survival (p <0.05). **(D)** Survival results for patients with different PD-1 expression levels: no difference was observed (p>0.05). **(E)** Survival outcome for 97 NSCLC patients with different PD-L1 expression levels. The survival rate of patients with a high PD-L1 expression level in cluster 3 was significantly increased (p<0.05). The median cutoff value was used to distinguish high density from low density. A log-rank test was used to perform statistical analysis.

We further evaluated the prognostic value of the peritumoral density levels of CD8+, FOXP3+, PD-1+, and PD-L1+ cells and the ratio of CD8/FOXP3, CD8/PD-1, and CD8/PD-L1 cells in three patient subgroups (i.e., cluster 1: medium CD8/FOXP3 ratio with high PD-1 and PD-L1 expression; cluster 2: low CD8/FOXP3 ratio with low PD-1 and PD-L1 expression; and cluster 3: high CD8/FOXP3 ratio with low PD-1 and PD-L1 expression) (**Figures 5A**–**E** and **Supplementary Figures S3A, B**). High levels of tumor-infiltrating CD8+ T cells are considered to be a good predictor of survival in many human cancer types (including NSCLC) (20). However, in our study, there was no significant correlation between tumor-infiltrating CD8+ T lymphocytes and patient survival in any patients or clusters **(** **Figures 4A**, **5A**). When evaluating the prognostic value of the CD8/FOXP3 ratio, a high CD8/FOXP3 ratio was found to predictably increase the patient survival rate in cluster 3 but not in other subgroups (**Figure 5B**). In contrast, the CD8/PD-1 ratio and CD8/PD-L1 ratio had no prognostic value in any patient subgroup (**Supplementary Figures S3A, B**). These results indicate that CD8 levels relative to FOXP3 levels have prognostic value in a subgroup of patients. In addition, patients in cluster 2 (low CD8/FOXP3 ratio) with high FOXP3 expression showed poor survival **(** **Figure 5C**), which may indicate that targeting FOXP3+ Treg cells can improve the function of CD8+ cells and benefit the outcomes of patients in this cluster. PD-1 expression showed no prognostic value in any of the 3 clusters (**Figure 5D**). Surprisingly, individual PD-L1 levels showed prognostic value in cluster 3 but not in cluster 1, and high PD-L1 expression was correlated with better survival outcomes (**Figure 5E**). These results may indicate that a high CD8/FOXP3 ratio with moderate PD-L1 expression can balance the effects of tumor reactivity and an immunosuppressive microenvironment, resulting in a better survival outcome.

### Immune Suppression Is Regulated by PD-1/PD-L1 in Association With EGFR Status

**Figure 6:**
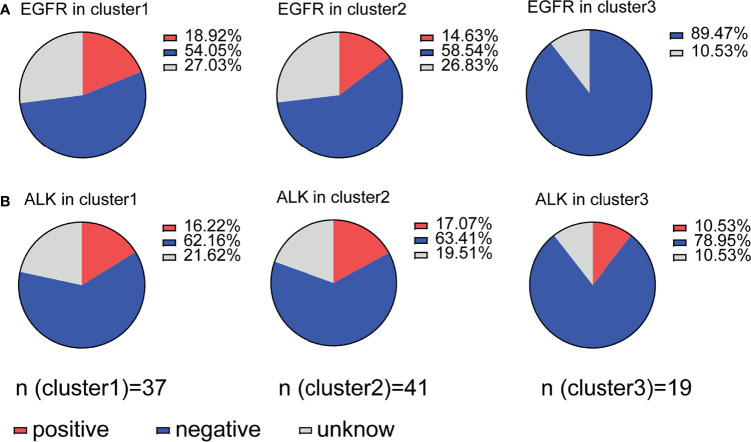
Association of immune suppression status with EGFR or ALK mutation. **(A)** Distribution of patients with EGFR mutations in clusters 1-3; clusters 1 and 2 (immune suppression) showed a relationship with mutation status, but cluster 3 did not. **(B)** Patients with ALK mutations were evenly distributed between all three clusters.

EGFR driver mutation-positive NSCLC tissues overexpress PD-L1 (21). There is a positive relationship between tumor PD-L1 expression and EGFR mutation status and poor prognosis in lung adenocarcinoma (22). We further evaluated whether immune suppression is regulated by PD-1/PDL-1 expression combined with EGFR or ALK mutation status. Cluster 1 (18.92%) and cluster 2 (14.63%) patients with immune suppression had more EGFR mutations than cluster 3 patients (0%) (**Figure 6A**), but ALK mutations did not have any effect in any of the three clusters: cluster 1 (16.22%), cluster 2 (17.07%) and cluster 3 (10.53%) (**Figure 6B**). Our data indicate that EGFR mutation status is associated with high TIL density and high PD-1/PD-L1 expression (cluster 1) and with low TIL density and low PD-1/PD-L1 expression (cluster 2). This result is in contrast with previous reports stating that NSCLC patients with EGFR mutations have high PD-L1 expression (23). We further evaluated the prognostic value of immune cells in gene-mutated patients and wild-type patients, and the results indicate that only CD8 levels relative to FOXP3 levels have prognostic value in a wild-type subgroup of patients (**Supplementary Figures S4A-G**), underlying the crucial prognostic value of the CD8/FOXP3 ratio.

### Establishment of an Immune Risk Score Model for Determining Prognosis Based on Multivariate Cox Regression

**Figure 7:**
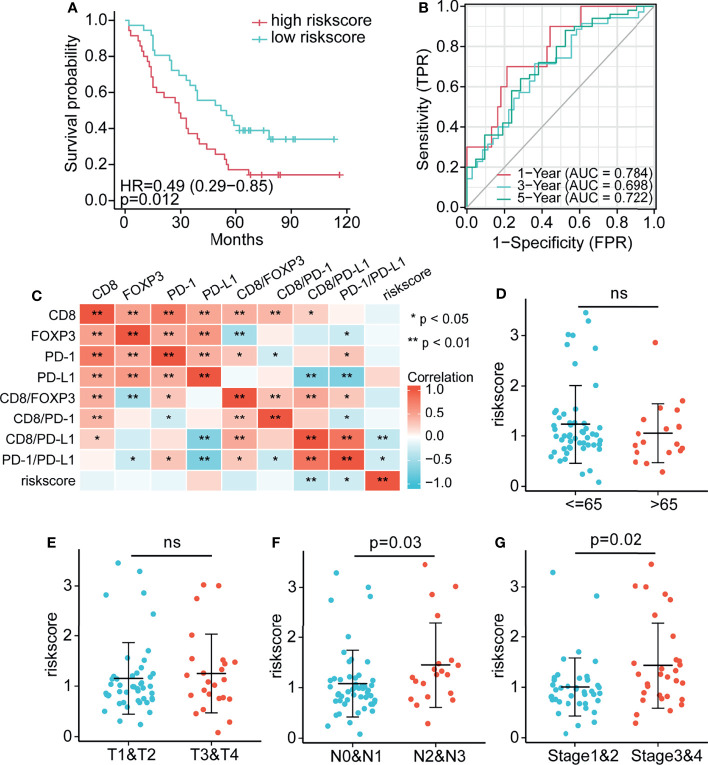
Establishment of the risk score and assessment of its prognostic significance **(A)** Compared with the high risk score group, the low risk score group had significantly better survival. **(B)** AUC values for 7-factor predictions models. **(C)** Heatmap comparing immune cell abundance with the risk score. **(D–G)** The association between clinical information, **(D)** age, **(E)** tumor, **(F)** lymph node, and **(G)** AJCC stage and the risk score. * p < 0.05, ** p < 0.01, ns p > 0.05.

Although immune clustering can predict the postoperative survival rate of NSCLC patients, it cannot provide a linear measurement of risk. Therefore, factors of interest were used to construct a risk score model: Risk score=0.391*CD8+ (-0.374) *FOXP3+(-0.396) *PD-1+0.272*PD-L1 +(-0.269) *CD8/FOXP3+(-0.473) *fCD8/PD-1+0.181*CD8/PD-L1 (**Supplementary Table S3**). Notably, the survival rate of patients with high risk scores was significantly lower than that of patients with low risk scores (P = 0.012; **Figure 7A**). The immune variables included in this score model are localized to peritumoral subregions, and therefore, the score reflects the prognosis of NSCLC patients based on the complex interaction of these components within peritumoral regions. In addition, to evaluate the sensitivity and specificity of the risk score in determining the prognosis of patients with NSCLC, a time-dependent ROC analysis was performed (**Figure 7B**). The results of ROC curve analysis showed that the 1-year, 3-year and 5-year OS AUC values were 0.784, 0.698 and 0.722, respectively, and models with higher AUC values showed a better performance than models with lower AUC values. Overall, the risk score model based on peritumoral immune marker levels was effective for prediction. **Figure 7C** shows the correlation heatmap between the immune cells and risk score. We further analyzed associations between clinicopathological characteristics (age, tumor stage, lymph node stage, and AJCC stage) and the risk score. **Figures 7D**–**G** shows that the risk scores of patients with higher lymph node stage (P = 0.03) and AJCC stage (P = 0.02) were significantly higher than those with lower lymph node stage and AJCC stage, indicating substantial prognostic value of the risk score model.

## Discussion

The evolution of cancer is greatly influenced by the cell type, cell density and location of immune cells in tumor subregions; immune checkpoints and tumor-associated immune cell distribution have positive and negative effects on patient prognosis (24). This study examined archived tumor specimens obtained from a cohort of patients with stage I–IV adenocarcinoma (NSCLC) using IHC. Image-based analysis of the expression of PD-L1 in malignant cells and the density of TILs expressing CD8, PD-1 and FOXP3 in intratumoral and peritumoral compartments was performed. The density of TILs expressing the immune markers of interest was significantly higher in the peritumoral area than in the intratumor compartment. Unsupervised hierarchical clustering analysis based on these markers (CD8/FOXP3, PD-1 and PD-L1) identified 3 clusters with significantly different survival, and a small number of patients with high CD8/FOXP3 and low PD-1 and PD-L1 immune checkpoint expression without EGFR mutation showed a better OS duration. Furthermore, a low CD8/FOXP3 ratio and high immune checkpoint expression may indicate a strong immune evasive ability of NSCLC. Based on the clustering results, stratification of patients might provide guidance for anti-PD-1/PD-L1 therapy; those with a lower CD8/FOXP3 ratio and lower immune checkpoint expression may benefit from strategies targeting Treg cells.

According to the density and distribution of FOXP3+ and CD8+ T cells etc, tumors can be classified as “hot” or “cold”, which can be used to predict the clinical outcome of various cancer patients. “Hot” indicates likely sensitivity to immune therapeutics (25). A comprehensive analysis of the type, density and location of immune cells in spatially distributed subregions may provide insights for the development of immunotherapy. Due to the heterogeneity in the distribution of the expression of the markers of interest, defined subregions with immune variables were analyzed individually for prognostic value. Our data showed that the CD8/FOXP3 ratio was higher in the peritumoral components than in the intratumoral components. These results indicate that the tumor is more likely to be immunosuppressed than the peritumor area, and this immunosuppressed status may affect different genetic characteristics of tumor cells. Recently, mutations in the enzyme cytosolic isocitrate dehydrogenase (IDH1) were discovered to inhibit STAT1 signaling to induce CD8+ T cell accumulation, thereby promoting immune evasion in gliomas (26). This finding partly explains why the intratumoral immune microenvironment is more susceptible to immunosuppression than the peritumoral immune microenvironment.

The tumor immune microenvironment is spatially heterogeneous, with differences being especially apparent between the tumor core and the infiltrating edge. Hepatocellular carcinoma (HCC) studies have reported that T cells, B cells and monocytes infiltrate the edge of the tumor core and are related to patient prognosis (27–29). The location of immune cells in CRC also has prognostic value that is superior to that of and independent of other prognostic factors and superior to traditional TNM staging (30). Consistent with this finding, it has been suggested that subregion-specific enrichment of immune cells is a promising prognostic factor for NSCLC patients (31). In addition, the density of immune cells has been shown to impact the immunotherapy response. For example, PD-1+ cells are potential biomarkers for anti-PD-1 immunotherapy in some cancer types, such as head and neck cancer and HCC (32, 33). Notably, we found that the distribution of PD-1+ cells among NSCLC subregions was highly heterogeneous, which could lead to different responses to PD-1 blockade. This idea is consistent with a recent report showing that the immune microenvironment is heterogeneous; in addition, the response to immunotherapy is often primary and is orchestrated by sophisticated tumor–host–microenvironment interactions in the peritumor and intratumor compartments.

Unsupervised hierarchical clustering analysis of the markers was used to define the correlation between CD8+ T cell, FOXP3+ Treg cell, PD-1+ cell and PD-L1 cell densities. We found that the density of FOXP3+, PD-1+ and PD-L1+ cells in tumor tissues increased when the density of CD8+ T cells was increased compared with that in adjacent normal tissues. Nonetheless, patient stratification based on each of these markers alone did not show significant prognostic value for predicting patient survival. A higher CD8/FOXP3 ratio is associated with a more favorable prognosis for esophageal, rectal, and head/neck cancer (34–36). However, to the best of our knowledge, there is no information on the use of the CD8/FOXP3 ratio in combination with the PD-1 and PD-L1 expression levels as a means for the stratification of patients with NSCLC. In addition, such combined consideration of the CD8/FOXP3 ratio and PD-1 and PD-L1 expression to predict patient cluster stratification may guide anti-PD-1/PD-L1 strategies or strategies targeting Treg cells for cancer immunotherapy. The clinically relevant threshold of IHC PD-L1 expression in NSCLC cells has not yet been established. Researchers have examined various cutoff values for PD-L1 positivity, including at least 5% (7, 9, 26), 10% (15), and 50% and greater than the median of the H score (37–39). However, the optimal cutoff values for PD-L1 and immune parameters have not yet been determined. Prospective studies using a larger number of patient samples will be required to standardize PD-L1 and immune parameters. Here, instead of using cutoffs of PD-L1 positivity, we used the CD8/FOXP3 ratio combined with both PD-1 and PD-L1 expression for stratification of NSCLC patients. The PD-1/PD-L1 pathway is the immune escape mechanism in 40% of cancers (40). Moreover, the tumor microenvironment, which includes tumor cells and TILs, has an important role in immunotherapy. FOXP3, a unique transcription factor, is specifically overexpressed in Treg cells and is used to identify Treg cells, which are mainly CD4+FOXP3+ T cells. Treg cells are generally thought to have immunosuppressive effects, but their effect on NSCLC prognosis is unclear. We hypothesize that the heterogeneous or spatially distributed secretion of cytokines and chemokines at the infiltrating tumor edge may promote the recruitment of different subtypes of TILs to tumor subregions (41). Curiously, in NSCLC, PD-L1 expression in malignant cells is significantly correlated with TIL density in the surrounding and intratumoral compartments. In different immune cell subpopulations, such as CD4+ and CD8+ T cells and natural killer (NK) cells, PD-L1 expression has been found to be induced in malignant cells *via* the production of IFNγ (42). Surprisingly, although we found a significant correlation between PD-L1 expression in malignant cells and PD-1 cell infiltration in NSCLC tumors, different immune resistance mechanisms were present among the different histological types of NSCLC around and inside the tumor environment. This was not in line with the mechanism in our research, and we characterized these two types of microenvironments (peritumoral and intratumoral compartments).

In this analysis, immune clustering based on immune cell density showed that patients could be divided into three subgroups with different immune cell distributions. Unsupervised hierarchical clustering analysis based on the markers of interest revealed that NSCLC patients with a higher CD8/FOXP3 ratio and low PD-1/PD-L1 immune checkpoint expression (cluster 3) and without EGFR mutation were likely to experience local disease control. PD-L1 overexpression is promoted by oncogenic and constitutive activation signals, including EGFR, Kirsten rat sarcoma virus oncogene homolog (KRAS), and protein kinase B (AKT), which are mechanisms of cell-intrinsic tumor resistance (21, 43, 44). PD-L1 can be induced in cancer and immune cells (myelosuppressive cells, dendritic cells, macrophages, and lymphocytes) in the tumor microenvironment through inflammatory signals. This mechanism is an example of adaptive resistance. On the other hand, a low CD8/FOXP3 ratio and high PD-1/PD-L1 expression (cluster 1) could reflect a stronger immune evasion ability of NSCLC tumors. Stratification of patients based on this clustering strategy might guide anti-PD-1/PD-L1 therapy and recommendation of Treg cell-targeting therapy for patients with a low CD8/FOXP3 ratio and PD-1/PD-L1 expression (cluster 2). Cox regression analysis was used to establish a risk score model considering immune variables in peritumoral subregions. Patients with a high risk score had higher lymph node stage and later stage disease, which may indicate that the prognosis of NSCLC patients depends on complex interactions in the peritumoral component. Since our immune risk score model was derived from the peritumoral compartment, it may not be appropriate to validate using a public database. However, we tried to use TCGA cohorts to validate our risk score model constructed from the TMA results (**Supplementary Figure S5**). The results were similar to the TMA data results, and high risk scores had a tendency to be correlated with poor survival outcomes (P=0.0558). These results also indicated that our model derived from the peritumoral compartment may have greater prognostic value (P=0.012). And maybe because the immune components are mainly located in the peritumoral compartment, TCGA cohort verification achieved matching results. Because the risk score model was derived from a mathematical algorithm, its biological significance needs to be defined. More immune subpopulations, such as macrophages, B cells, and dendritic cells, must be evaluated to gain insight into the heterogeneous immune microenvironment in malignant tumors and to illustrate clinical relevance.

Selecting patient biomarkers for therapies has been difficult. PD-L1 expression detected by immunohistochemistry is a possible biomarker. To date, the available data show some conflicting results, but PD-L1 immunohistochemistry seems likely to be introduced into the clinic to select patients for anti-PD-1 or anti-PD-L1 treatment (45). Given that four drugs are rapidly approaching regulatory approval and each drug has its own independent PD-L1 immunohistochemical biomarker test, oncologists and pathologists are facing some major challenges. The biology of the PD-1/PD-L1 axis is complex, clinical anti-PD-1/PD-L1 treatment has shown considerable variation, and the selectivity of PD-L1 biomarker detection is not perfect (46). Based on our cluster models for classification, stratification of patients based on the clustering results might guide anti-PD-1/PD-L1 or Treg targeting cancer immunotherapy. The cluster characteristics might also serve as promising prognostic predictors for NSCLC patients. Furthermore, our future studies will focus on the highly heterogeneous microenvironment and explore the spatial distribution of the expression of CD8, FOXP3, PD-1, PD-L1, and other immune checkpoints to better stratify patients and guide clinical immunotherapy for NSCLC patients.

## Conclusions

In conclusion, through clustering analysis based on markers of interest, we showed that a higher CD8/FOXP3 ratio and low PD-1 and PD-L1 immune checkpoint expression combined with a lack of EGFR mutation could be a favorable predictive marker for local control. We performed clustering based on the CD8/FOXP3 ratio, PD-1 and PD-L1 immune checkpoint expression and stratified patients into 3 clusters with different prognostic markers to guide cancer immunotherapy (anti-PD-1/PD-L1 therapy or targeting of Treg cells). In addition, based on these markers, we established a risk score model with value to determine the prognosis of NSCLC patients.

## Data Availability Statement

The original contributions presented in the study are included in the article/**Supplementary Material**. Further inquiries can be directed to the corresponding authors.

## Ethics Statement

The studies involving human participants were reviewed and approved by Shanghai Jiao Tong University Human Ethics Committees. The patients/participants provided their written informed consent to participate in this study.

## Author Contributions

GJ and YY contributed to the concept and designed the research. YY, XY, YW, JX, HS, HG, and XQ performed the experiments and acquired, analyzed and interpreted the data. YY, XY, GJ, and YW drafted the article. All authors have read and agreed to the published version of the manuscript.

## Funding

This work was supported by grants from the National Natural Science Foundation of China (NSFC) (81500060) and the Pilot Project of Clinical Collaboration between Traditional Chinese and Western Medicine (grant ZXYXZ-201901).

## Conflict of Interest

The authors declare that the research was conducted in the absence of any commercial or financial relationships that could be construed as a potential conflict of interest.

## Publisher’s Note

All claims expressed in this article are solely those of the authors and do not necessarily represent those of their affiliated organizations, or those of the publisher, the editors and the reviewers. Any product that may be evaluated in this article, or claim that may be made by its manufacturer, is not guaranteed or endorsed by the publisher.

## References

[1] Toumazis I Bastani M Han SS Plevritis SK. Risk-Based Lung Cancer Screening: A Systematic Review. Lung Cancer (2020) 147:154–86. doi: 10.1016/j.lungcan.2020.07.007 32721652

[2] Rotow J Bivona TG. Understanding and Targeting Resistance Mechanisms in NSCLC. Nat Rev Cancer (2017) 17(11):637–58. doi: 10.1038/nrc.2017.84 29068003

[3] Iams WT Porter J Horn L. Immunotherapeutic Approaches for Small-Cell Lung Cancer. Nat Rev Clin Oncol (2020) 17(5):300–12. doi: 10.1038/s41571-019-0316-z PMC7212527 32055013

[4] Saab S Zalzale H Rahal Z Khalifeh Y Sinjab A Kadara H. Insights Into Lung Cancer Immune-Based Biology, Prevention, and Treatment. Front Immunol (2020) 11:159. doi: 10.3389/fimmu.2020.00159 32117295 PMC7026250

[5] Topper MJ Vaz M Chiappinelli KB DeStefano Shields CE Niknafs N Yen RC. Epigenetic Therapy Ties MYC Depletion to Reversing Immune Evasion and Treating Lung Cancer. Cell (2017) 171(6):1284–300 e21. doi: 10.1016/j.cell.2017.10.022 29195073 PMC5808406

[6] Horvath L Thienpont B Zhao L Wolf D Pircher A. Overcoming Immunotherapy Resistance in non-Small Cell Lung Cancer (NSCLC) - Novel Approaches and Future Outlook. Mol Cancer (2020) 19(1):141. doi: 10.1186/s12943-020-01260-z 32917214 PMC7488475

[7] Zhang R Chen C Dong X Shen S Lai L He J. Independent Validation of Early-Stage Non-Small Cell Lung Cancer Prognostic Scores Incorporating Epigenetic and Transcriptional Biomarkers With Gene-Gene Interactions and Main Effects. Chest (2020) 158(2):808–19. doi: 10.1016/j.chest.2020.01.048 PMC7417380 32113923

[8] Lizotte PH Ivanova EV Awad MM Jones RE Keogh L Liu H. Multiparametric Profiling of non-Small-Cell Lung Cancers Reveals Distinct Immunophenotypes. JCI Insight (2016) 1(14):e89014. doi: 10.1172/jci.insight.89014 27699239 PMC5033841

[9] Obeid JM Wages NA Hu Y Deacon DH Slingluff CL Jr. Heterogeneity of CD8(+) Tumor-Infiltrating Lymphocytes in Non-Small-Cell Lung Cancer: Impact on Patient Prognostic Assessments and Comparison of Quantification by Different Sampling Strategies. Cancer Immunol Immunother: CII (2017) 66(1):33–43. doi: 10.1007/s00262-016-1908-4 27770170 PMC5512540

[10] Boulle G Velut Y Mansuet-Lupo A Gibault L Blons H Fournel L. Chemoradiotherapy Efficacy is Predicted by Intra-Tumour CD8+/FoxP3+ Double Positive T Cell Density in Locally Advanced N2 Non-Small-Cell Lung Carcinoma. Eur J Cancer (2020) 135:221–9. doi: 10.1016/j.ejca.2020.04.040 32610210

[11] Ying L Yan F Meng Q Yu L Yuan X Gantier MP. PD-L1 Expression is a Prognostic Factor in Subgroups of Gastric Cancer Patients Stratified According to Their Levels of CD8 and FOXP3 Immune Markers. Oncoimmunology (2018) 7(6):e1433520. doi: 10.1080/2162402X.2018.1433520 29872566 PMC5980489

[12] Miyashita M Sasano H Tamaki K Hirakawa H Takahashi Y Nakagawa S. Prognostic Significance of Tumor-Infiltrating CD8+ and FOXP3+ Lymphocytes in Residual Tumors and Alterations in These Parameters After Neoadjuvant Chemotherapy in Triple-Negative Breast Cancer: A Retrospective Multicenter Study. Breast Cancer Res: BCR (2015) 17:124. doi: 10.1186/s13058-015-0632-x 26341640 PMC4560879

[13] Teng MW Ngiow SF Ribas A Smyth MJ. Classifying Cancers Based on T-Cell Infiltration and PD-L1. Cancer Res (2015) 75(11):2139–45. doi: 10.1158/0008-5472.CAN-15-0255 PMC4452411 25977340

[14] Tokito T Azuma K Kawahara A Ishii H Yamada K Matsuo N. Predictive Relevance of PD-L1 Expression Combined With CD8+ TIL Density in Stage III non-Small Cell Lung Cancer Patients Receiving Concurrent Chemoradiotherapy. Eur J Cancer (2016) 55:7–14. doi: 10.1016/j.ejca.2015.11.020 26771872

[15] Hofman P Badoual C Henderson F Berland L Hamila M Long-Mira E. Multiplexed Immunohistochemistry for Molecular and Immune Profiling in Lung Cancer-Just About Ready for Prime-Time? Cancers (2019) 11(3):283. doi: 10.3390/cancers11030283 PMC6468415 30818873

[16] Giraldo NA Nguyen P Engle EL Kaunitz GJ Cottrell TR Berry S. Multidimensional, Quantitative Assessment of PD-1/PD-L1 Expression in Patients With Merkel Cell Carcinoma and Association With Response to Pembrolizumab. J Immunother Cancer (2018) 6(1):99. doi: 10.1186/s40425-018-0404-0 30285852 PMC6167897

[17] Toth ZE Mezey E. Simultaneous Visualization of Multiple Antigens With Tyramide Signal Amplification Using Antibodies From the Same Species. J Histochem Cytochem: Off J Histochem Soc (2007) 55(6):545–54. doi: 10.1369/jhc.6A7134.2007 17242468

[18] Brody R Zhang Y Ballas M Siddiqui MK Gupta P Barker C. PD-L1 Expression in Advanced NSCLC: Insights Into Risk Stratification and Treatment Selection From a Systematic Literature Review. Lung Cancer (2017) 112:200–15. doi: 10.1016/j.lungcan.2017.08.005 29191596

[19] Li B Cui Y Nambiar DK Sunwoo JB Li R. The Immune Subtypes and Landscape of Squamous Cell Carcinoma. Clin Cancer Res: an Off J Am Assoc Cancer Res (2019) 25(12):3528–37. doi: 10.1158/1078-0432.CCR-18-4085 PMC6571041 30833271

[20] Thommen DS Koelzer VH Herzig P Roller A Trefny M Dimeloe S. A Transcriptionally and Functionally Distinct PD-1(+) CD8(+) T Cell Pool With Predictive Potential in Non-Small-Cell Lung Cancer Treated With PD-1 Blockade. Nat Med (2018) 24(7):994–1004. doi: 10.1038/s41591-018-0057-z 29892065 PMC6110381

[21] Azuma K Ota K Kawahara A Hattori S Iwama E Harada T. Association of PD-L1 Overexpression With Activating EGFR Mutations in Surgically Resected Nonsmall-Cell Lung Cancer. Ann Oncol: Off J Eur Soc Med Oncol (2014) 25(10):1935–40. doi: 10.1093/annonc/mdu242 25009014

[22] Inamura K Yokouchi Y Sakakibara R Kobayashi M Subat S Ninomiya H. Relationship of Tumor PD-L1 Expression With EGFR Wild-Type Status and Poor Prognosis in Lung Adenocarcinoma. Japanese J Clin Oncol (2016) 46(10):935–41. doi: 10.1093/jjco/hyw087 27511990

[23] Masuda K Horinouchi H Tanaka M Higashiyama R Shinno Y Sato J. Efficacy of Anti-PD-1 Antibodies in NSCLC Patients With an EGFR Mutation and High PD-L1 Expression. J Cancer Res Clin Oncol (2021) 147(1):245–51. doi: 10.1007/s00432-020-03329-0 PMC7810613 32705363

[24] Galon J Bruni D. Approaches to Treat Immune Hot, Altered and Cold Tumours With Combination Immunotherapies. Nat Rev Drug Discov (2019) 18(3):197–218. doi: 10.1038/s41573-018-0007-y 30610226

[25] Taube JM. Unleashing the Immune System: PD-1 and PD-Ls in the Pre-Treatment Tumor Microenvironment and Correlation With Response to PD-1/PD-L1 Blockade. Oncoimmunology (2014) 3(11):e963413. doi: 10.4161/21624011.2014.963413 25914862 PMC4292419

[26] Kohanbash G Carrera DA Shrivastav S Ahn BJ Jahan N Mazor T. Isocitrate Dehydrogenase Mutations Suppress STAT1 and CD8+ T Cell Accumulation in Gliomas. J Clin Invest (2017) 127(4):1425–37. doi: 10.1172/JCI90644 PMC5373859 28319047

[27] Gabrielson A Wu Y Wang H Jiang J Kallakury B Gatalica Z. Intratumoral CD3 and CD8 T-Cell Densities Associated With Relapse-Free Survival in HCC. Cancer Immunol Res (2016) 4(5):419–30. doi: 10.1158/2326-6066.CIR-15-0110 PMC5303359 26968206

[28] Liu LZ Zhang Z Zheng BH Shi Y Duan M Ma LJ. CCL15 Recruits Suppressive Monocytes to Facilitate Immune Escape and Disease Progression in Hepatocellular Carcinoma. Hepatology (2019) 69(1):143–59. doi: 10.1002/hep.30134 30070719

[29] Chen DP Ning WR Li XF Wei Y Lao XM Wang JC. Peritumoral Monocytes Induce Cancer Cell Autophagy to Facilitate the Progression of Human Hepatocellular Carcinoma. Autophagy (2018) 14(8):1335–46. doi: 10.1080/15548627.2018.1474994 PMC6103724 29940792

[30] Galon J Costes A Sanchez-Cabo F Kirilovsky A Mlecnik B Lagorce-Pages C. Type, Density, and Location of Immune Cells Within Human Colorectal Tumors Predict Clinical Outcome. Science (2006) 313(5795):1960–4. doi: 10.1126/science.1129139 17008531

[31] Camy F Karpathiou G Dumollard JM Magne N Perrot JL Vassal F. Brain Metastasis PD-L1 and CD8 Expression is Dependent on Primary Tumor Type and its PD-L1 and CD8 Status. J Immunother Cancer (2020) 8(2):e000597. doi: 10.1136/jitc-2020-000597 32859740 PMC7454240

[32] Cohen EEW Bell RB Bifulco CB Burtness B Gillison ML Harrington KJ. The Society for Immunotherapy of Cancer Consensus Statement on Immunotherapy for the Treatment of Squamous Cell Carcinoma of the Head and Neck (HNSCC). J Immunother Cancer (2019) 7(1):184. doi: 10.1186/s40425-019-0662-5 31307547 PMC6632213

[33] Sangro B Sarobe P Hervas-Stubbs S Melero I. Advances in Immunotherapy for Hepatocellular Carcinoma. Nat Rev Gastroenterol Hepatol (2021) 18(8):525–43. doi: 10.1038/s41575-021-00438-0 PMC8042636 33850328

[34] Zhu Y Li M Mu D Kong L Zhang J Zhao F. CD8+/FOXP3+ Ratio and PD-L1 Expression Associated With Survival in Pt3n0m0 Stage Esophageal Squamous Cell Cancer. Oncotarget (2016) 7(44):71455–65. doi: 10.18632/oncotarget.12213 PMC5342092 27683115

[35] Spector ME Bellile E Amlani L Zarins K Smith J Brenner JC. Prognostic Value of Tumor-Infiltrating Lymphocytes in Head and Neck Squamous Cell Carcinoma. JAMA Otolaryngol Head Neck Surg (2019) 145(11):1012–9. doi: 10.1001/jamaoto.2019.2427 PMC6735419 31486841

[36] Shinto E Hase K Hashiguchi Y Sekizawa A Ueno H Shikina A. CD8+ and FOXP3+ Tumor-Infiltrating T Cells Before and After Chemoradiotherapy for Rectal Cancer. Ann Surg Oncol (2014) 21(Suppl 3):S414–21. doi: 10.1245/s10434-014-3584-y 24566864

[37] Teixido C Vilarino N Reyes R Reguart N. PD-L1 Expression Testing in non-Small Cell Lung Cancer. Ther Adv Med Oncol (2018) 10:1758835918763493. doi: 10.1177/1758835918763493 29662547 PMC5898658

[38] Forde PM Chaft JE Smith KN Anagnostou V Cottrell TR Hellmann MD. Neoadjuvant PD-1 Blockade in Resectable Lung Cancer. N Engl J Med (2018) 378(21):1976–86. doi: 10.1056/NEJMoa1716078 PMC6223617 29658848

[39] Aguilar EJ Ricciuti B Gainor JF Kehl KL Kravets S Dahlberg S. Outcomes to First-Line Pembrolizumab in Patients With Non-Small-Cell Lung Cancer and Very High PD-L1 Expression. Ann Oncol: Off J Eur Soc Med Oncol (2019) 30(10):1653–9. doi: 10.1093/annonc/mdz288 31435660

[40] Yarchoan M Hopkins A Jaffee EM. Tumor Mutational Burden and Response Rate to PD-1 Inhibition. N Engl J Med (2017) 377(25):2500–1. doi: 10.1056/NEJMc1713444 PMC6549688 29262275

[41] Parra ER Behrens C Rodriguez-Canales J Lin H Mino B Blando J. Image Analysis-Based Assessment of PD-L1 and Tumor-Associated Immune Cells Density Supports Distinct Intratumoral Microenvironment Groups in Non-Small Cell Lung Carcinoma Patients. Clin Cancer Res: an Off J Am Assoc Cancer Res (2016) 22(24):6278–89. doi: 10.1158/1078-0432.CCR-15-2443 PMC5558040 27252415

[42] Waldman AD Fritz JM Lenardo MJ. A Guide to Cancer Immunotherapy: From T Cell Basic Science to Clinical Practice. Nat Rev Immunol (2020) 20(11):651–68. doi: 10.1038/s41577-020-0306-5 PMC7238960 32433532

[43] Li S Li L Zhu Y Huang C Qin Y Liu H. Coexistence of EGFR With KRAS, or BRAF, or PIK3CA Somatic Mutations in Lung Cancer: A Comprehensive Mutation Profiling From 5125 Chinese Cohorts. Br J Cancer (2014) 110(11):2812–20. doi: 10.1038/bjc.2014.210 PMC4037826 24743704

[44] Chen N Fang W Zhan J Hong S Tang Y Kang S. Upregulation of PD-L1 by EGFR Activation Mediates the Immune Escape in EGFR-Driven NSCLC: Implication for Optional Immune Targeted Therapy for NSCLC Patients With EGFR Mutation. J Thorac Oncol: Off Publ Int Assoc Study Lung Cancer (2015) 10(6):910–23. doi: 10.1097/JTO.0000000000000500 25658629

[45] Ancevski Hunter K Socinski MA Villaruz LC. PD-L1 Testing in Guiding Patient Selection for PD-1/PD-L1 Inhibitor Therapy in Lung Cancer. Mol Diagnos Ther (2018) 22(1):1–10. doi: 10.1007/s40291-017-0308-6 PMC5773410 29119407

[46] Kerr KM Nicolson MC. Non-Small Cell Lung Cancer, PD-L1, and the Pathologist. Arch Pathol Lab Med (2016) 140(3):249–54. doi: 10.5858/arpa.2015-0303-SA 26927720

