# Supplementary figures and images for: Combined Consideration of Tumor-Associated Immune Cell Density and Immune Checkpoint Expression in the Peritumoral Microenvironment for Prognostic Stratification of Non-Small-Cell Lung Cancer Patients
Source: Front Immunol. 2022 Feb 10;13:811007. doi: 10.3389/fimmu.2022.811007 (PMC8866234; doi:10.3389/fimmu.2022.811007)

**Supplementary Figure S1**


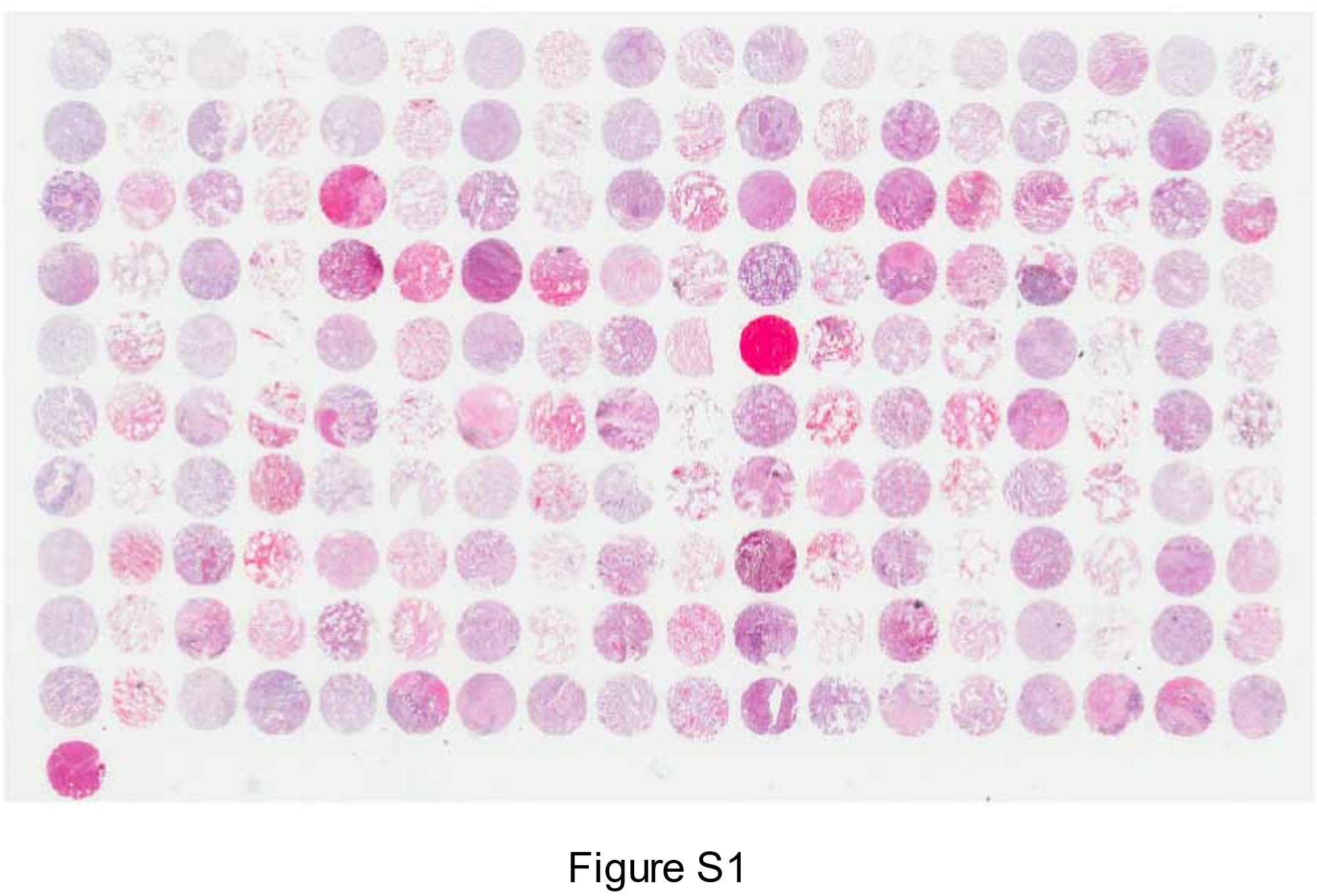


**Figure S1. H&E results of the 180-core TMA.**

Supplement: Supplementary file 1 [file DataSheet_1.docx]
