# Supplementary material for: Combined Consideration of Tumor-Associated Immune Cell Density and Immune Checkpoint Expression in the Peritumoral Microenvironment for Prognostic Stratification of Non-Small-Cell Lung Cancer Patients
Source: Front Immunol. 2022 Feb 10;13:811007. doi: 10.3389/fimmu.2022.811007 (PMC8866234; doi:10.3389/fimmu.2022.811007)
Supplement: Supplementary file 2 [file DataSheet_2.docx]

**Supplementary Figure S2**


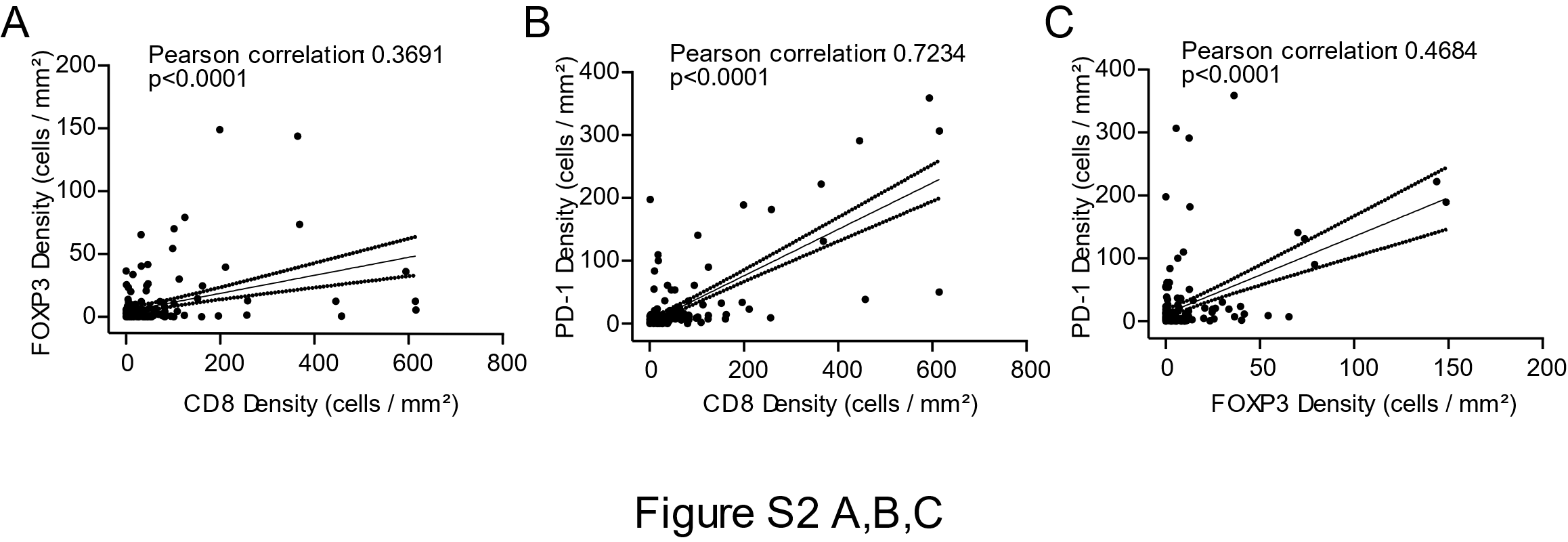


**Figure S2.** Pearson correlation between densities of **(A)** CD8 and FOXP3, **(B)** CD8 and PD-1, **(C)** FOXP3 and PD-1 (n=97 for tumor tissue samples).
