# Supplementary material for: Combined Consideration of Tumor-Associated Immune Cell Density and Immune Checkpoint Expression in the Peritumoral Microenvironment for Prognostic Stratification of Non-Small-Cell Lung Cancer Patients
Source: Front Immunol. 2022 Feb 10;13:811007. doi: 10.3389/fimmu.2022.811007 (PMC8866234; doi:10.3389/fimmu.2022.811007)
Supplement: Supplementary file 3 [file DataSheet_3.docx]

**Supplementary Figure S3**


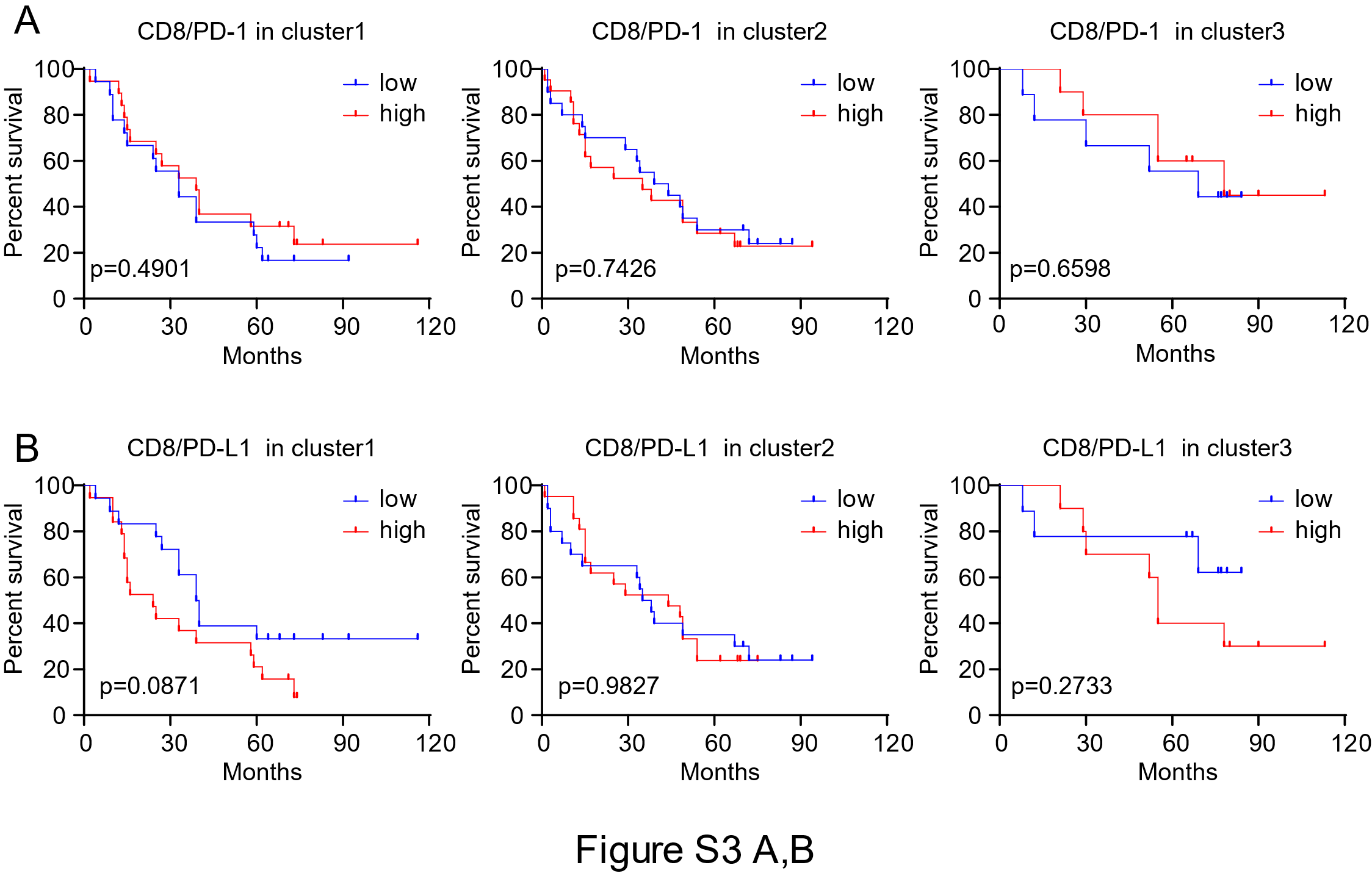


**Figure S3.** Kaplan-Meier analyses of overall survival (OS) for **(A)** CD8/PD-1 and **(B)** CD8/PD-L1 in 3 clusters generated according to their CD8/FOXP3 ratio, PD-1 and PD-L1 densities. A median cutoff was used to separate high and low populations. Log rank test was used to determine significance.
