# Supplementary material for: Combined Consideration of Tumor-Associated Immune Cell Density and Immune Checkpoint Expression in the Peritumoral Microenvironment for Prognostic Stratification of Non-Small-Cell Lung Cancer Patients
Source: Front Immunol. 2022 Feb 10;13:811007. doi: 10.3389/fimmu.2022.811007 (PMC8866234; doi:10.3389/fimmu.2022.811007)
Supplement: Supplementary file 4 [file DataSheet_4.docx]

**Supplementary Figure S4**


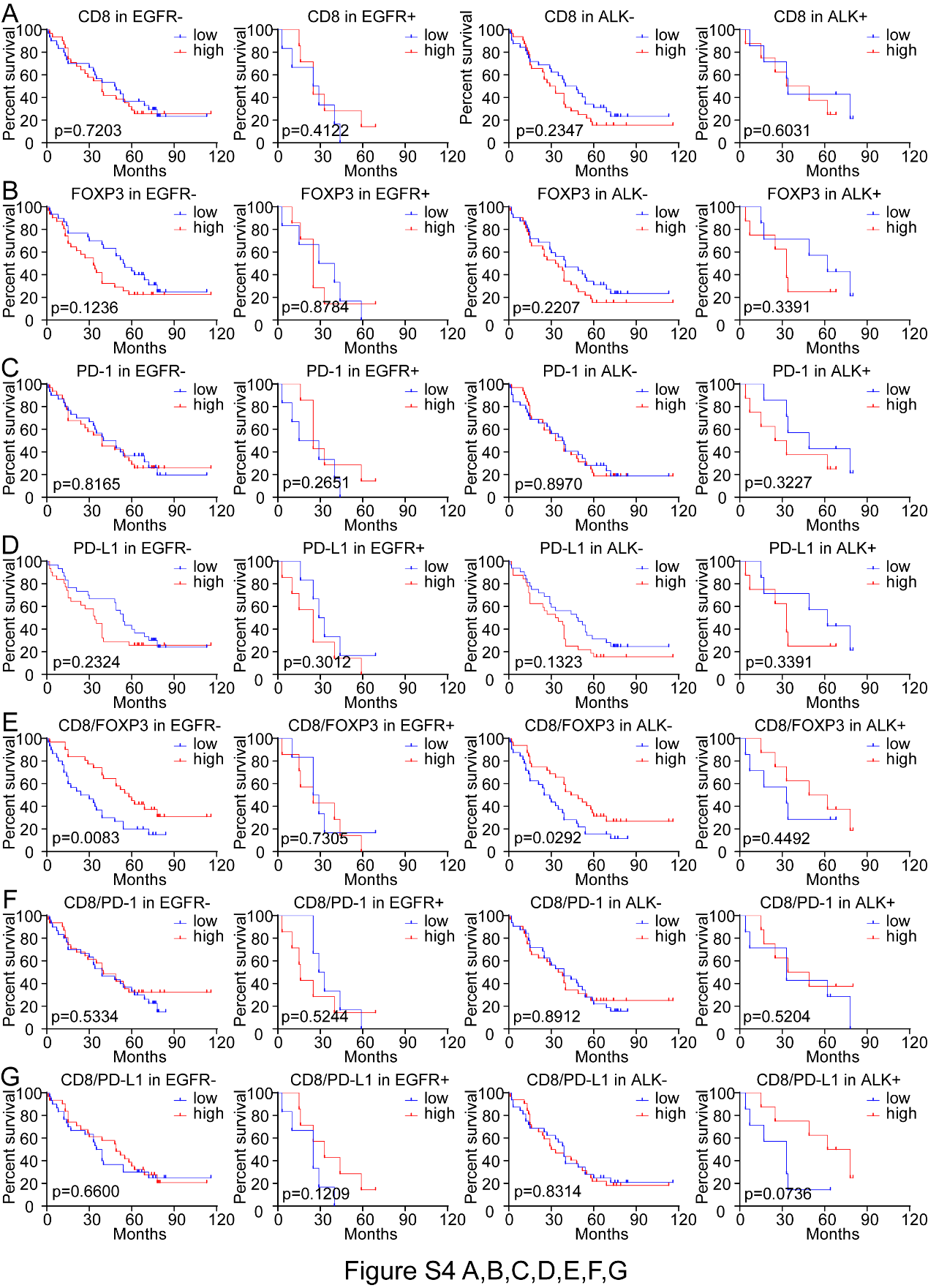


**Figure S4.** Kaplan-Meier analyses of overall survival (OS) for **(A)** CD8, **(B)** FOXP3, **(C)** PD-1, **(D)** PD-L1, **(E)** CD8/FOXP3, **(F)** CD8/PD-1, **(G)** CD8/PD-L1 in EGFR or ALK gene-mutated and wild-type patients. A median cutoff was used to separate high and low populations. Log rank test was used to determine significance.
