# Supplementary material for: Combined Consideration of Tumor-Associated Immune Cell Density and Immune Checkpoint Expression in the Peritumoral Microenvironment for Prognostic Stratification of Non-Small-Cell Lung Cancer Patients
Source: Front Immunol. 2022 Feb 10;13:811007. doi: 10.3389/fimmu.2022.811007 (PMC8866234; doi:10.3389/fimmu.2022.811007)
Supplement: Supplementary file 5 [file DataSheet_5.docx]

**Supplementary Figure S5**


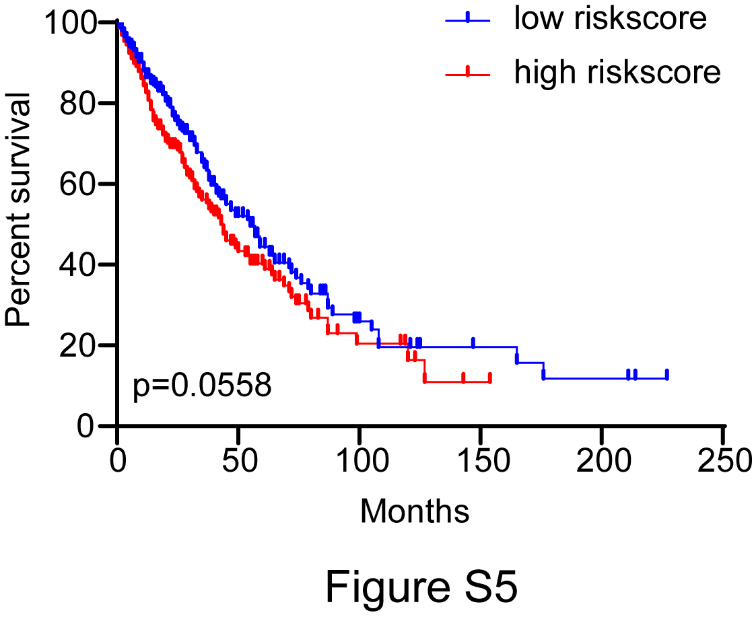


**Figure S5.** Kaplan-Meier analysis of overall survival (OS) in TCGA cohort to validate the risk score model constructed from the TMA results.
