# Supplementary material for: Combined Consideration of Tumor-Associated Immune Cell Density and Immune Checkpoint Expression in the Peritumoral Microenvironment for Prognostic Stratification of Non-Small-Cell Lung Cancer Patients
Source: Front Immunol. 2022 Feb 10;13:811007. doi: 10.3389/fimmu.2022.811007 (PMC8866234; doi:10.3389/fimmu.2022.811007)
Supplement: Supplementary file 6 [file Table_1.docx]

**Table S1. Patients’ information.**

| No. | Tissues | Gender | Age | T | N | M | Clinical stage | Grade | Survival months | Status |
| --- | --- | --- | --- | --- | --- | --- | --- | --- | --- | --- |
| 1 | tumor | F | 71 | T1b | Nx | M0 | 2-3 | 2 | 69 | deceased |
| 2 | tumor | M | 47 | T2a | N1 | M0 | 2A | 2 | 94 | survival |
| 3 | tumor | M | 74 | T1b | N0 | M0 | 1A | 2 | 78 | deceased |
| 4 | tumor | M | 58 | T3 | N1 | M0 | 3A | 2 | 38 | deceased |
| 5 | tumor | M | 30 | T2a | Nx | M0 | 2-3 | 2 | 49 | deceased |
| 6 | tumor | M | 67 | T2b | N0 | M0 | 2A | 3 | 92 | survival |
| 7 | tumor | M | 57 | T1b | N0 | M0 | 1A | 2 | 116 | survival |
| 8 | tumor | F | 25 | T3 | N0 | M0 | 2B | 1-2 | 90 | survival |
| 9 | tumor | F | 64 | T1b | N0 | M0 | 1A | 2 | 113 | survival |
| 10 | tumor | M | 50 | T2a | N0 | M0 | 1B | 2-3 | 87 | survival |
| 11 | tumor | M | 57 | T2a | Nx | M0 | 2-3 | 2 | 33 | deceased |
| 12 | tumor | F | 50 | T2a | N0 | M0 | 1B | 2 | 21 | deceased |
| 13 | tumor | F | 46 | T1b | N0 | M0 | 1A | 2-3 | 83 | survival |
| 14 | tumor | F | 72 | T2a | N0 | M0 | 1B | 2-3 | 39 | deceased |
| 15 | tumor | F | 55 | T1b | N0 | M0 | 1A | 2-3 | 34 | deceased |
| 16 | tumor | F | 50 | T2b | Nx | M0 | 2-3 | 3 | 39 | deceased |
| 17 | tumor | M | 60 | T2a | N0 | M0 | 1B | 2-3 | 11 | deceased |
| 18 | tumor | M | 47 | — | Nx | M0 | 2-3 | 3 | 73 | deceased |
| 19 | tumor | M | 65 | T2a | N0 | M0 | 1B | 2 | 15 | deceased |
| 20 | tumor | F | 58 | T3 | N1 | M0 | 3A | 1 | 55 | deceased |
| 21 | tumor | F | 67 | T3 | N1 | M0 | 3A | 3 | 10 | deceased |
| 22 | tumor | F | 68 | T2a | N0 | M0 | 1B | 2 | 62 | deceased |
| 23 | tumor | M | 55 | T3 | N1 | M0 | 3A | 3 | 33 | deceased |
| 24 | tumor | F | 59 | T3 | N2 | M0 | 3A | 3 | 14 | deceased |
| 25 | tumor | F | 56 | T2a | N0 | M1b | 4 | 2-3 | 49 | deceased |
| 26 | tumor | F | 62 | T2a | N1 | M0 | 2A | 2 | 13 | deceased |
| 27 | tumor | M | 74 | T1b | N0 | M0 | 1A | 2 | 72 | survival |
| 28 | tumor | F | 76 | T1b | N0 | M0 | 1A | 1 | 71 | survival |
| 29 | tumor | M | 49 | — | Nx | M0 | 2-3 | 2 | 70 | survival |
| 30 | tumor | M | 75 | T1b | N0 | M0 | 1A | 2 | 4 | deceased |
| 31 | tumor | F | 69 | T1b | N0 | M0 | 1A | 2 | 11 | deceased |
| 32 | tumor | F | 52 | T2a | N0 | M0 | 1B | 2-3 | 60 | deceased |
| 33 | tumor | M | 55 | T1b | N0 | M0 | 1A | 2-3 | 15 | deceased |
| 34 | tumor | M | 65 | T1 | N0 | M0 | 1A | 2 | 68 | survival |
| 35 | tumor | F | 66 | T3 | N3 | M0 | 3B | 2-3 | 9 | deceased |
| 36 | tumor | M | 42 | T3 | N1 | M0 | 3A |  | 10 | deceased |
| 37 | tumor | M | 53 | T2a | N2 | M0 | 3A | 2 | 17 | deceased |
| 38 | tumor | M | 66 | T2b | N1 | M0 | 2B | 2 | 33 | deceased |
| 39 | tumor | F | 57 | T2a | N1 | M0 | 2A | 2 | 59 | deceased |
| 40 | tumor | F | 51 | T2a | Nx | M0 | 2-3 | 1-2 | 48 | deceased |
| 41 | tumor | F | 65 | T4 | N2 | M0 | 3B | 2-3 | 3 | deceased |
| 42 | tumor | M | 64 | T4 | N0 | M0 | 3A | 2 | 27 | deceased |
| 43 | tumor | M | 71 | T3 | N3 | M0 | 3B | 2-3 | 44 | deceased |
| 44 | tumor | F | 60 | T2a | N0 | M0 | 1B | 2-3 | 54 | deceased |
| 45 | tumor | M | 61 | T2a | N1 | M0 | 2A | 2 | 25 | deceased |
| 46 | tumor | M | 60 | T2a | N0 | M0 | 1B | 2 | 77 | survival |
| 47 | tumor | F | 58 | T2a | N0 | M0 | 1B | 2 | 67 | deceased |
| 48 | tumor | M | 60 | T1a | N2 | M0 | 3A | 2 | 29 | deceased |
| 49 | tumor | M | 63 | T2a | N2 | M0 | 3A | 2 | 16 | deceased |
| 50 | tumor | F | 77 | T2a | N0 | M0 | 1B | 1 | 62 | survival |
| 51 | tumor | F | 81 | T1b | Nx | M0 | 2-3 | 2 | 13 | deceased |
| 52 | tumor | M | 61 | T2a | Nx | M0 | 2-3 | 2 | 40 | deceased |
| 53 | tumor | M | 84 | T1b | N0 | M0 | 1A | 2 | 84 | survival |
| 54 | tumor | M | 65 | T2a | Nx | M0 | 2-3 | 2-3 | 76 | survival |
| 55 | tumor | M | 53 | T2a | N1 | M0 | 2A | 2-3 | 83 | survival |
| 56 | tumor | M | 49 | T3 | N1 | M0 | 3A | 2 | 33 | deceased |
| 57 | tumor | M | 74 | T3 | N3 | M0 | 3B | 2 | 40 | deceased |
| 58 | tumor | M | 64 | T2b | N3 | M0 | 3B | 2-3 | 15 | deceased |
| 59 | tumor | F | 73 | T3 | N0 | M0 | 2B | 1-2 | 80 | survival |
| 60 | tumor | M | 52 | T1a | Nx | M0 | 2-3 | 2 | 72 | deceased |
| 61 | tumor | M | 44 | T3 | N0 | M0 | 2B | 1 | 35 | deceased |
| 62 | tumor | M | 55 | T3 | N1 | M0 | 3A | 2 | 69 | survival |
| 63 | tumor | F | 50 | T3 | N2 | M0 | 3A | 2 | 25 | deceased |
| 64 | tumor | M | 78 | T2a | N1 | M0 | 2A | 2 | 49 | deceased |
| 65 | tumor | F | 60 | T2a | N0 | M0 | 1B | 2-3 | 39 | deceased |
| 66 | tumor | M | 54 | T2a | N1 | M0 | 2A | 2 | 58 | deceased |
| 67 | tumor | M | 66 | T2b | N0 | M0 | 2A | 3 | 7 | deceased |
| 68 | tumor | F | 54 | T2a | N2 | M0 | 3A | 2-3 | 75 | survival |
| 69 | tumor | F | 48 | T1a | — | M0 | — | 2 | 1 | deceased |
| 70 | tumor | M | 59 | T2a | N1 | M0 | 2A | 2 | 74 | survival |
| 71 | tumor | M | 58 | T2a | N0 | M0 | 1B | 3 | 52 | deceased |
| 72 | tumor | F | 56 | T4 | N2 | M0 | 3B | 2 | 54 | deceased |
| 73 | tumor | F | 53 | T2a | N2 | M0 | 3A | 2 | 14 | deceased |
| 74 | tumor | F | 62 | T2b | N2 | M0 | 3A | 2-3 | 12 | deceased |
| 75 | tumor | M | 72 | T2b | Nx | M0 | 2-3 | 2 | 12 | deceased |
| 76 | tumor | F | 71 | T1b | Nx | M0 | 2-3 | 2 | 69 | deceased |
| 77 | tumor | M | 61 | T3 | Nx | M0 | 3 | 2 | 3 | deceased |
| 78 | tumor | F | 65 | T1a | N0 | M0 | 1A | 2 | 15 | deceased |
| 79 | tumor | F | 53 | T2b | N0 | M0 | 2A | 2 | 2 | deceased |
| 80 | tumor | F | 67 | T4 | N2 | M0 | 3B | 2-3 | 2 | deceased |
| 81 | tumor | M | 65 | T3 | N1 | M0 | 3A | 2 | 29 | deceased |
| 82 | tumor | F | 66 | T3 | N0 | M0 | 2B | 2 | 68 | survival |
| 83 | tumor | M | 74 | T2a | N3 | M0 | 3B | 3 | 2 | deceased |
| 84 | tumor | F | 20 | T1b | N0 | M0 | 1A | 2 | 39 | deceased |
| 85 | tumor | M | 51 | T2a | Nx | M0 | 2-3 | 2 | 25 | deceased |
| 86 | tumor | M | 73 | T2a | N0 | M0 | 1B | 3 | 68 | survival |
| 87 | tumor | F | 57 | T4 | N2 | M0 | 3B | 2 | 15 | deceased |
| 88 | tumor | M | 75 | T2a | N0 | M0 | 1B | 1-2 | 67 | survival |
| 89 | tumor | M | 60 | T1b | Nx | M0 | 2-3 | 2 | 10 | deceased |
| 90 | tumor | M | 36 | T2a | N2 | M0 | 3A | 2 | 30 | deceased |
| 91 | tumor | M | 74 | T2b | N0 | M0 | 2A | 1-2 | 24 | deceased |
| 92 | tumor | F | 51 | T2b | N0 | M0 | 2A | 2-3 | 65 | survival |
| 93 | tumor | F | 58 | T2a | N0 | M0 | 1B | 2 | 55 | deceased |
| 94 | tumor | F | 62 | T3 | N2 | M0 | 3A | 3 | 8 | deceased |
| 95 | tumor | F | 73 | T3 | N0 | M0 | 2B | 3 | 64 | survival |
| 96 | tumor | M | 59 | T3 | N3 | M0 | 3B | 2 | 79 | survival |
| 97 | tumor | M | 65 | T2a | N0 | M0 | 1B | 3 | 73 | survival |
| 98 | normal | M | 61 | T2a | N0 | M0 | 1B | 2 | 14 | deceased |
| 99 | normal | M | 63 | T2a | N1 | M0 | 2A | 3 | 6 | deceased |
| 100 | normal | M | 47 | T2a | N1 | M0 | 2A | 2 | 94 | survival |
| 101 | normal | M | 74 | T1b | N0 | M0 | 1A | 2 | 78 | deceased |
| 102 | normal | M | 58 | T3 | N1 | M0 | 3A | 2 | 38 | deceased |
| 103 | normal | M | 30 | T2a | Nx | M0 | 2-3 | 2 | 49 | deceased |
| 104 | normal | M | 67 | T2b | N0 | M0 | 2A | 3 | 92 | survival |
| 105 | normal | M | 57 | T1b | N0 | M0 | 1A | 2 | 116 | survival |
| 106 | normal | F | 25 | T3 | N0 | M0 | 2B | 1-2 | 90 | survival |
| 107 | normal | F | 64 | T1b | N0 | M0 | 1A | 2 | 113 | survival |
| 108 | normal | M | 50 | T2a | N0 | M0 | 1B | 2-3 | 87 | survival |
| 109 | normal | M | 57 | T2a | Nx | M0 | 2-3 | 2 | 33 | deceased |
| 110 | normal | F | 72 | T2a | N0 | M0 | 1B | 2-3 | 39 | deceased |
| 111 | normal | F | 55 | T1b | N0 | M0 | 1A | 2-3 | 34 | deceased |
| 112 | normal | F | 50 | T2b | Nx | M0 | 2-3 | 3 | 39 | deceased |
| 113 | normal | M | 60 | T2a | N0 | M0 | 1B | 2-3 | 11 | deceased |
| 114 | normal | M | 47 | — | Nx | M0 | 2-3 | 3 | 73 | deceased |
| 115 | normal | M | 65 | T2a | N0 | M0 | 1B | 2 | 15 | deceased |
| 116 | normal | F | 58 | T3 | N1 | M0 | 3A | 1 | 55 | deceased |
| 117 | normal | F | 67 | T3 | N1 | M0 | 3A | 3 | 10 | deceased |
| 118 | normal | F | 68 | T2a | N0 | M0 | 1B | 2 | 62 | deceased |
| 119 | normal | M | 55 | T3 | N1 | M0 | 3A | 3 | 33 | deceased |
| 120 | normal | F | 59 | T3 | N2 | M0 | 3A | 3 | 14 | deceased |
| 121 | normal | F | 56 | T2a | N0 | M1b | 4 | 2-3 | 49 | deceased |
| 122 | normal | F | 62 | T2a | N1 | M0 | 2A | 2 | 13 | deceased |
| 123 | normal | M | 74 | T1b | N0 | M0 | 1A | 2 | 72 | survival |
| 124 | normal | F | 76 | T1b | N0 | M0 | 1A | 1 | 71 | survival |
| 125 | normal | M | 75 | T1b | N0 | M0 | 1A | 2 | 4 | deceased |
| 126 | normal | F | 69 | T1b | N0 | M0 | 1A | 2 | 11 | deceased |
| 127 | normal | F | 52 | T2a | N0 | M0 | 1B | 2-3 | 60 | deceased |
| 128 | normal | M | 55 | T1b | N0 | M0 | 1A | 2-3 | 15 | deceased |
| 129 | normal | M | 65 | T1 | N0 | M0 | 1A | 2 | 68 | survival |
| 130 | normal | F | 66 | T3 | N3 | M0 | 3B | 2-3 | 9 | deceased |
| 131 | normal | M | 42 | T3 | N1 | M0 | 3A | 2 | 10 | deceased |
| 132 | normal | M | 53 | T2a | N2 | M0 | 3A | 2 | 17 | deceased |
| 133 | normal | F | 57 | T2a | N1 | M0 | 2A | 2 | 59 | deceased |
| 134 | normal | F | 51 | T2a | Nx | M0 | 2-3 | 1-2 | 48 | deceased |
| 135 | normal | M | 71 | T3 | N3 | M0 | 3B | 2-3 | 44 | deceased |
| 136 | normal | F | 60 | T2a | N0 | M0 | 1B | 2-3 | 54 | deceased |
| 137 | normal | M | 61 | T2a | N1 | M0 | 2A | 2 | 25 | deceased |
| 138 | normal | M | 63 | T2a | N2 | M0 | 3A | 2 | 16 | deceased |
| 139 | normal | F | 81 | T1b | Nx | M0 | 2-3 | 2 | 13 | deceased |
| 140 | normal | M | 61 | T2a | Nx | M0 | 2-3 | 2 | 40 | deceased |
| 141 | normal | M | 65 | T2a | Nx | M0 | 2-3 | 2-3 | 76 | survival |
| 142 | normal | M | 53 | T2a | N1 | M0 | 2A | 2-3 | 83 | survival |
| 143 | normal | M | 49 | T3 | N1 | M0 | 3A | 2 | 33 | deceased |
| 144 | normal | M | 74 | T3 | N3 | M0 | 3B | 2 | 40 | deceased |
| 145 | normal | M | 64 | T2b | N3 | M0 | 3B | 2-3 | 15 | deceased |
| 146 | normal | M | 44 | T3 | N0 | M0 | 2B | 1 | 35 | deceased |
| 147 | normal | M | 55 | T3 | N1 | M0 | 3A | 2 | 69 | survival |
| 148 | normal | F | 50 | T3 | N2 | M0 | 3A | 2 | 25 | deceased |
| 149 | normal | M | 78 | T2a | N1 | M0 | 2A | 2 | 49 | deceased |
| 150 | normal | F | 60 | T2a | N0 | M0 | 1B | 2-3 | 39 | deceased |
| 151 | normal | M | 54 | T2a | N1 | M0 | 2A | 2 | 58 | deceased |
| 152 | normal | M | 66 | T2b | N0 | M0 | 2A | 3 | 7 | deceased |
| 153 | normal | F | 54 | T2a | N2 | M0 | 3A | 2-3 | 75 | survival |
| 154 | normal | F | 48 | T1a | — | M0 | — | 2 | 1 | deceased |
| 155 | normal | M | 59 | T2a | N1 | M0 | 2A | 2 | 74 | survival |
| 156 | normal | M | 58 | T2a | N0 | M0 | 1B | 3 | 52 | deceased |
| 157 | normal | F | 56 | T4 | N2 | M0 | 3B | 2 | 54 | deceased |
| 158 | normal | F | 53 | T2a | N2 | M0 | 3A | 2 | 14 | deceased |
| 159 | normal | F | 62 | T2b | N2 | M0 | 3A | 2-3 | 12 | deceased |
| 160 | normal | M | 61 | T3 | Nx | M0 | 3 | 2 | 3 | deceased |
| 161 | normal | F | 65 | T1a | N0 | M0 | 1A | 2 | 15 | deceased |
| 162 | normal | F | 53 | T2b | N0 | M0 | 2A | 2 | 2 | deceased |
| 163 | normal | F | 67 | T4 | N2 | M0 | 3B | 2-3 | 2 | deceased |
| 164 | normal | M | 65 | T3 | N1 | M0 | 3A | 2 | 29 | deceased |
| 165 | normal | F | 66 | T3 | N0 | M0 | 2B | 2 | 68 | survival |
| 166 | normal | F | 20 | T1b | N0 | M0 | 1A | 2 | 39 | deceased |
| 167 | normal | M | 51 | T2a | Nx | M0 | 2-3 | 2 | 25 | deceased |
| 168 | normal | M | 73 | T2a | N0 | M0 | 1B | 3 | 68 | survival |
| 169 | normal | F | 57 | T4 | N2 | M0 | 3B | 2 | 15 | deceased |
| 170 | normal | M | 75 | T2a | N0 | M0 | 1B | 1-2 | 67 | survival |
| 171 | normal | M | 60 | T1b | Nx | M0 | 2-3 | 2 | 10 | deceased |
| 172 | normal | M | 36 | T2a | N2 | M0 | 3A | 2 | 30 | deceased |
| 173 | normal | M | 74 | T2b | N0 | M0 | 2A | 1-2 | 24 | deceased |
| 174 | normal | F | 51 | T2b | N0 | M0 | 2A | 2-3 | 65 | survival |
| 175 | normal | F | 58 | T2a | N0 | M0 | 1B | 2 | 55 | deceased |
| 176 | normal | F | 73 | T3 | N0 | M0 | 2B | 3 | 64 | survival |
| 177 | normal | M | 65 | T2a | N0 | M0 | 1B | 3 | 73 | survival |
| 178 | normal | F | 77 | T2a | N0 | M0 | 1B | 1 | 62 | survival |
|  |  |  |  |  |  |  |  |  |  |  |
