# Supplementary material for: Combined Consideration of Tumor-Associated Immune Cell Density and Immune Checkpoint Expression in the Peritumoral Microenvironment for Prognostic Stratification of Non-Small-Cell Lung Cancer Patients
Source: Front Immunol. 2022 Feb 10;13:811007. doi: 10.3389/fimmu.2022.811007 (PMC8866234; doi:10.3389/fimmu.2022.811007)
Supplement: Supplementary file 7 [file Table_2.docx]

**Table S2. Characteristics of patients.**

| **Parameter** | **Stratification** | **Number of patients** |
| --- | --- | --- |
| **Gender** | **Male** | **54** |
|  | **Female** | **44** |
| **Age** | **＜60** | **45** |
|  | **≥60** | **53** |
| **Status** | **Deceased** | **71** |
|  | **Survival** | **27** |
| **Tumor** | **T1** | **21** |
|  | **T2** | **49** |
|  | **T3** | **21** |
|  | **T4** | **5** |
|  | **—** | **2** |
| **Lymph Node** | **N0** | **44** |
|  | **N1** | **17** |
|  | **N2** | **14** |
|  | **N3** | **6** |
|  | **Nx** | **16** |
|  | **—** | **2** |
| **Metastasis** | **M0** | **97** |
|  | **M1b** | **1** |
| **Clinical Stage** | **1** | **32** |
|  | **2** | **19** |
|  | **2-3** | **15** |
|  | **3** | **30** |
|  | **4** | **1** |
|  | **—** | **1** |
| **Grade** | **1** | **4** |
|  | **1-2** | **5** |
|  | **2** | **55** |
|  | **2-3** | **20** |
|  | **3** | **13** |
|  | **—** | **1** |
