# Supplementary material for: Combined Consideration of Tumor-Associated Immune Cell Density and Immune Checkpoint Expression in the Peritumoral Microenvironment for Prognostic Stratification of Non-Small-Cell Lung Cancer Patients
Source: Front Immunol. 2022 Feb 10;13:811007. doi: 10.3389/fimmu.2022.811007 (PMC8866234; doi:10.3389/fimmu.2022.811007)
Supplement: Supplementary file 8 [file Table_3.docx]

**Table S3. Seven parameters were selected out to establish the risk score.**

|  | coef | exp(coef) | se(coef) | z | Pr(>\|z\|) |
| --- | --- | --- | --- | --- | --- |
| CD8 | 0.391 | 1.479 | 0.253 | 1.546 | 0.122 |
| FOXP3 | -0.374 | 0.688 | 0.159 | -2.352 | 0.019 |
| PD-1 | -0.396 | 0.673 | 0.263 | -1.504 | 0.133 |
| PD-L1 | 0.272 | 1.312 | 0.093 | 2.928 | 0.003 |
| CD8/FOXP3 | -0.269 | 0.764 | 0.110 | -2.449 | 0.014 |
| CD8/PD-1 | -0.473 | 0.623 | 0.310 | -1.526 | 0.127 |
| CD8/PD-L1 | 0.181 | 1.199 | 0.076 | 2.389 | 0.017 |
